# Platelet plug microstructure and flow modulate fibrin gelation dynamics: Insights from computational simulations

**Published:** 2026-04-09

**Authors:** Janneke M.H. Cruts, Frank J.H. Gijsen, Aaron L. Fogelson, Anna C. Nelson

**Affiliations:** 1 Department of Biomedical Engineering, Erasmus Medical Center, Rotterdam, the Netherlands; 2 Department of Biomechanical Engineering, Delft University of Technology, Delft, the Netherlands; 3 Department of Mathematics, University of Utah, Salt Lake City, Utah, United States; 4 Department of Biomedical Engineering, University of Utah, Salt Lake City, Utah, United States; 5 Department of Mathematics and Statistics, University of New Mexico, Albuquerque, New Mexico, United States

**Keywords:** blood clot formation, thrombus, fibrin polymerization, platelets, mathematical modeling

## Abstract

During the formation of a thrombus, the architecture of the growing platelet aggregate is heterogeneous, with areas of dense and loosely packed platelets. The surface of activated platelets facilitate biochemical coagulation reactions that ultimately result in the formation of a fibrin network which stabilizes the thrombus. How platelet-plug microstructure and flow jointly govern the onset and development of fibrin is incompletely understood. We developed a novel 2D computational framework that integrates (1) a pre-adhered, discrete platelet aggregate, (2) a reduced coagulation model that generates thrombin, and (3) a fibrin polymerization model. Three platelet-plug configurations were constructed with prescribed interplatelet gaps and simulations were performed for each with various wall shear rates. We quantified spatiotemporal clotting metrics, including coagulation factor concentrations, fibrin evolution, and gelation onset. Across geometries, gelation initiation accelerated with increasing plug density. For more dense geometries, gelation emerged first near the plug periphery. As the platelet density increased, intraplug transport was increasingly restricted, particularly for fibrinogen and prothrombin, and the thrombin concentrations in the spaces between platelets increased. In contrast, the loose plug supported fibrinogen replenishment deeper into the plug core. Thus, despite slower coagulation initiation due to reduced platelet surface area, monomer generation persisted further into the interior, causing gelation to begin at the vessel wall instead of the periphery. For all plug densities, fibrin ultimately filled the plug within the simulated time frame, while increasing shear reduced the gel-covered area outside the plug. These results suggest a mechanistic tradeoff: rapid sealing of the injured vessel wall by early platelet contraction, i.e. plug densification, may impede the intraplug fibrin formation needed for durable stabilization. The proposed model provides a basis for systematic studies of platelet–coagulation interactions under flow, including extensions toward therapeutic developments relevant to prevention of cardiovascular disease.

## Author summary

We present a mathematical model that incorporates three processes which comprise the early stages of blood clot formation: the generation of the enzyme thrombin through coagulation reactions, the formation of a stabilizing fibrin gel, and a preformed platelet aggregate which facilitates coagulation reactions on discrete surfaces. While previous mathematical models have been developed to understand each of these three processes individually, few have investigated them simultaneously while incorporating platelet structure heterogeneity and varying flow conditions. Our results suggest that early fibrin gelation dynamics are influenced heavily by both flow and intraplug architecture, which each affect transport within the aggregate. By treating platelets as discrete surfaces rather than a continuum, we find that chemical accumulation and transport within the preformed platelet aggregate determines the location of the initial fibrin gel. Our simulations show that a dense clot results in a fibrin gel that first appears on the outer edge of the platelet aggregate, with thrombin trapped within the platelet plug interior. For the loose plug the fibrin gel first appeared close to the injured wall. These findings suggest a hypothesis as to why clot contraction occurs later in time.

## 2 Introduction

In the event of an injury to the blood vessel wall, exposed material in the subendothelium (SE) triggers the formation of a blood clot, or thrombus, which grows and covers the injury to prevent further bleeding. This hemostatic response to injury involves important biophysical and biochemical processes which includes platelet aggregation and coagulation [1, 2]. Platelets are small, disc-shaped cellular components of blood which become activated when they contact collagen exposed on the injured vessel wall. These platelets can adhere to the vessel wall and release soluble platelet agonists that activate nearby platelets allowing them to cohere to the wall-adherent platelets and to form a platelet plug. The surface of activated platelets supports essential biochemical reactions of the blood coagulation network which is a large system of enzymatic reactions that occur on the injured vessel wall, the surfaces of activated platelets, and in the fluid near these platelets. Coagulation involves many tightly regulated positive and negative feedback loops with various inhibitors to localize enzyme production, and results in the production of the enzyme thrombin. Thrombin has many functions, including activation of platelets and conversion of the blood-soluble protein fibrinogen into insoluble fibrin monomers that polymerize to form a space-filling, fibrin mesh that stabilizes the growing platelet plug.

The intertwined processes of platelet aggregation, coagulation, and fibrin polymerization occur under a wide range of flow conditions, with shear rates ranging from < 200 s^−1^ to up to 20, 000 s^−1^ in venous and arterial environments, respectively [3]. The development and structure of the growing thrombus can strongly affect the surrounding flow field, and consequently, affect the transport of proteins to, from and within the growing thrombus. These, in turn, impact clot formation, determining how quickly a clot forms and whether it remains intact. Experimental evidence indicates that a growing thrombus is heterogeneous, with a dense inner core of activated platelets able to facilitate coagulation reactions and a less dense shell of aggregated platelets and fibrin polymer [4, 5, 6, 7]. Within this microstructured environment, the conventional understanding of coagulation is that thrombin and subsequently fibrin are generated through both the extrinsic pathway, initiated by tissue factor (TF) exposure, and the intrinsic pathway of coagulation. These are essential for stabilizing the growing thrombus [8]. Platelet-mediated coagulation and fibrin generation typically begin adjacent to the vessel wall, near the TF source, and subsequently propagate outward through and beyond the platelet plug [1, 9, 10]. Additionally, there exists threshold behavior for thrombus initiation, characterized by minimal clot formation at low TF levels and rapid thrombin generation above a critical TF concentration [11, 12]. However, the spatial pattern of fibrin formation can be influenced by several factors, including the composition of the injured vessel surface, the local flow conditions (shear rate), and the evolving platelet microstructure [9]. The dense packing of activated platelets creates a physical barrier, resulting in restricted access for plasma proteins to the core of this platelet plug [4]. Furthermore, as the thrombus matures, a fibrin cap may form on its surface, imposing an additional transport barrier. This cap spatially separates the procoagulant platelet core from the flowing blood, which contains inactive coagulation factors and platelets [13]. The size of this fibrin cap has been shown to increase as the local shear rate decreases [9]. Despite these observations, the exact nature of the interaction between the evolving platelet plug microstructure and the shear rate in affecting the spatiotemporal development of fibrin remains an area requiring further investigation.

Many spatiotemporal computational and mathematical models have been proposed to study aspects of thrombus formation with or without fluid transport [3, 14, 15, 16, 17, 18, 19]. Mathematical frameworks have also been developed to study fibrin polymerization on various temporal and spatial scales, where continuum and discrete, molecular dynamics approaches have been utilized to gain multiscale insight into fibrin gel formation; we refer the reader to [17, 18] for reviews on mathematical models of fibrin polymerization. Several of these models make a continuum assumption and use ordinary (ODEs) or partial (PDEs) differential equations to study individual components of the clotting system. Studies on how the coagulation cascade is influenced by flow-mediated transport and platelet surface availability have provided mechanistic insight into various pathological states [20, 21, 22, 23, 24]. However, these studies do not include fibrin polymerization and assume that the platelet aggregate is a continuum so clot microstructure is not studied. Despite this, computational studies [22] qualitatively agree with experimental studies that show development of a clot with a core-shell structure that limits transport into and out of the core [4]. To understand the clot microstructure and the impact of discrete platelet surfaces, some mathematical models of thrombus formation use a multiscale framework. Here, platelets in the microscale are assumed to be discrete structures within a cellular Potts model [25] or use a Dissipating Particle Dynamics (DPD) framework to describe some volume of fluid or platelet aggregate, where coagulation reactions in the macroscale are described by diffusion-advection-reaction PDEs [26].

While many mathematical studies have focused on individual parts of blood clot formation, few have incorporated coagulation reactions in the fluid and on preformed platelet surfaces with fibrin polymerization and fluid transport within, into, and out of the aggregate. Some computational models have focused on understanding the biomechanics and stability of preformed, continuum clots comprised of a dense core of procoagulant platelets and fibrin mesh with a less dense shell of aggregated platelets [27]. Others have been interested in studying the transport of solutes within continuum, preformed clots with an impermeable platelet core and a boundary layer of fixed thrombin concentration; thrombin can then transport through a permeable loose fibrin cap [28]. Recent studies have investigated variation in the clot microstructure to understand transport into, within, and out of the discrete platelet plug [29, 30, 31], showing that the densely-packed inner core traps thrombin in the intraclot gaps so solute concentrations may increase [30] and diffusive transport decreases [31]. Recent modeling efforts have also incorporated machine learning techniques to provide patient-specific simulations of clot microstructure formation to predict thrombus growth [29, 32, 33].

Here, we seek to understand how variation in platelet microstructure can impact fibrin polymerization and thus the formation of a fibrin mesh. In particular, we seek to gain insight into how the coagulation reactions, which occur both in the fluid and on platelet surfaces, are affected by changes in the platelet microstructure. Toward this end, we develop a novel two-dimensional computational model with platelet plugs comprised of discrete platelets with a prescribed platelet packing density and we determine flow and protein transport under physiologically relevant shear conditions. This study systematically explores how platelet microstructure and shear rate together modulate thrombin generation and fibrin polymerization. By quantifying the temporal and spatial metrics of clot initiation, such as thrombin concentration, fibrin development, and gelation onset, we aim to provide deeper mechanistic insights into the initial steps of thrombus formation.

## 3 Materials and methods

**Fig 1.**
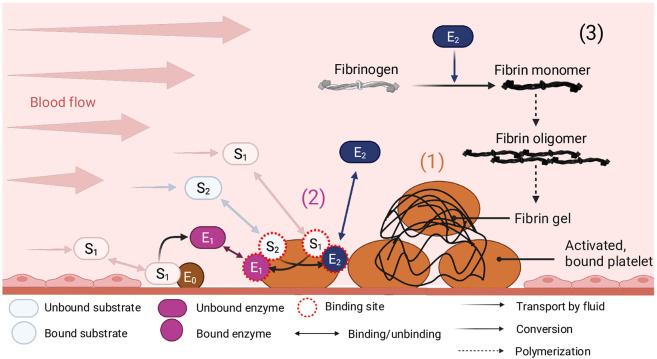
Schematic of mathematical framework depicting (1) the discrete platelet plug, the (2) reduced coagulation model of thrombin generation on the surfaces of platelets in the plug, and (3) the fibrin polymerization model which assumes species larger than monomer are not transported. We envision that the variables of the reduced model of coagulation roughly represent actual coagulation species $E_{0}$ (TF:fVIIa), $S_{1}$ (fX), $E_{1}$ (fXa), $S_{2}$ (prothrombin), $E_{2}$ (thrombin), $C_{2}^{b}=S_{1}^{b}\;:E_{2}^{b}$ (platelet-bound tenase fVIIIa:fIXa), and $C_{1}^{b}=S_{2}^{b}\;:E_{1}^{b}$ (platelet-bound prothrombinase). Here, dashed arrows represent polymerization reactions, tapered arrows represent transport, and conversion is shown with a faded arrow. Binding and unbinding of species is indicated with a double-ended arrow. Here, fluid-phase species are shown as a capsule shape, bound species are circles, and zymogens and enzymes are represented with light and dark hued shapes, respectively.

Our two-dimensional modeling framework consists of three main components that can affect or be affected by flow conditions: (1) a preformed platelet plug that is comprised of discrete platelets, where each activated platelet’s surface contains specified numbers of binding sites for coagulation proteins; (2) a reduced model of coagulation adapted from [34] that produces the enzyme thrombin; and (3) a model of fibrin polymerization similar to [35, 36] where fibrinogen monomer is sourced upstream and is converted by thrombin to fibrin monomers that can polymerize into a fibrin gel. A schematic of these three model components under flow is shown in Figure 1. This discrete-continuum modeling framework includes discrete platelets with surface concentrations of platelet-bound protein species and volumetric concentrations of other fluid-phase species. The reduced model of coagulation contains reactions among several biochemical species that are categorized as fluid-phase, platelet-bound, or subendothelium-bound. It is adapted from our more comprehensive model [11]. Finally, the mathematical description of fibrin polymerization tracks over space and time the concentrations of individual fibrin monomers $c_{10}$, monomers in oligomers θ, branches B, free reactive sites R, and a function Y that indicates progress toward gelation [35, 36]. In the subsections below and in the supplement, we describe each modeling component, and we provide parameter values used in the model in Table 1.

The model uses a continuum description of fluid, coagulation factors, and fibrin species. We regard the blood as an incompressible Newtonian fluid that is governed by the Navier-Stokes equation:

(1) $$\rho \left(\frac{\partial u}{\partial t}+(u\cdot \nabla )u\right)=-\nabla p+\mu \nabla^{2}u,$$

(2) ∇⋅u=0.

where the unknowns are the fluid velocity u(x,t) and fluid pressure p(x,t), and the values for the density ρ and dynamic viscosity μ are given in Table 1. Unlike some other models that use a Darcy term in the fluid dynamics equations to model the platelet plug as a porous material [22, 27, 37], here the fluid flows in the gaps between discrete stationary platelets and the presence of the platelets directly affects the fluid motion through the requirement that the fluid velocity be zero at each point of a platelet surface. A parabolic inlet velocity profile is specified as appropriate to generate flows with wall shear rates of 0 s^−1^ (no-flow), 100 s^−1^, or 1000 s^−1^. A constant outlet pressure of zero is specified, and no-slip boundary conditions are applied on the portions of the domain boundary representing blood vessel walls. Flow is allowed to reach a steady-state solution before beginning to simulate the coagulation and polymerization models.

### Discrete platelet model

The platelets are modeled as discrete, stationary disks with locations specified from the start of a simulation. Initially, each platelet becomes activated at a rate $k_{\text{act}}$. In the model, the effect of activation is to make binding sites on the platelet surfaces available for coagulation proteins. These binding sites are essential for the enzymatic reactions on those surfaces. There are two classes of binding sites, *P*_1_ for substrate $S_{1}$ and enzyme $E_{1}$, and *P*_2_ for substrate $S_{2}$ and enzyme $E_{2}$. The maximum number densities of the binding sites on an activated platelet are denoted $N_{1}^{\max}$ and $N_{2}^{\max}$, respectively (see Table 1). With t measured from the start of a simulation, the number density of type i binding sites at time t is

(3) $$N_{i}(t)=N_{i}^{\max}(1-e^{-k_{\text{act}}t}).$$

**Fig 2.**
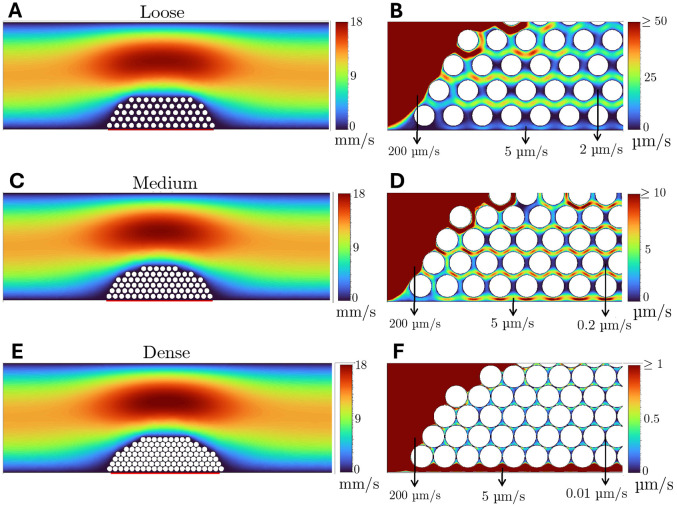
Velocity profiles for each platelet plug for an initial wall shear rate of 1000 s^−1^. Whole domain velocity profiles for (A) loose, (C) medium, and (E) dense platelet plug geometry. Velocity profiles for intraplug velocities for (B) loose, (D) medium, and (F) dense platelet plug geometries. Colors represent computed velocity values and note the difference in color bar ranges/units for each subfigure. The red strip of wall below the platelet aggregate represents a 50 μm length segment of the vessel wall where $E_{0}$ is nonzero. Note the differences in velocity ranges for (B), (D), and (F).

The binding sites are thus not available instantaneously, they increase gradually and approach their maximum over time. With $k_{\text{act}}=0.05$, 95% of binding sites become available within about 60 s. As illustrated in Figs. 2A,C,E, we constructed three distinct pre-adhered platelet plug geometries within a two-dimensional rectangular channel which represents a blood vessel. The geometries differed in the uniform interplatelet gap size used; 0.1 μm (dense), 0.5 μm (medium), and 1 μm (loose). The platelets have diameter 2.46 μm and a consistent spacing of 0.5 μm is maintained between the lowest row of platelets and the bottom vessel wall. The platelet plug geometries are determined to achieve consistent overall dimensions, forming a half ellipse shape with maximum height of 17 μm and width of 54 μm. Table 2 contains the characteristics of each geometry. Each platelet arrangement was generated using LAMMPS molecular dynamics software, and was subsequently meshed in Ansys Mechanical, where a general body element size of 2 μm is specified for use far from the platelets and refined near the platelets to conform with a mesh element size of 0.1 μm for platelet surfaces and 1 μm for the lower wall surface.

### Reduced coagulation model

The coagulation reaction system is complex with multiple enzyme activation steps, feedback reactions, and inhibitors. Previous models, including our own [15, 16, 22, 23, 24], have incorporated up to approximately 140 species. Here, we use a reduced model which we believe adequately captures the salient features of thrombin production for our current purposes. Importantly, the reduced model involves biochemical species that can be subendothelium-bound (SE-bound), platelet-bound, or fluid-phase. The critical reactions happen on the subendothelial surface or platelet surfaces, and products of reactions on one surface must move through the fluid to other surfaces to participate in other reactions. So, diffusive and advective transport play important roles in providing substrates for the surface-phase enzyme reactions, allowing enzymes produced on one surface to move to a second surface, and in carrying fluid-phase enzymes out of the platelet aggregate and then downstream. The model we use is adapted from that of Montgomery et al. [34] and is similar to the earlier model of Fogelson and Kuharsky [20].

A schematic of the reduced model reactions is given in Figure 1. The model involves enzyme $E_{0}$ on the SE that roughly corresponds to the TF:fVIIa complex. It forms a complex with fluid-phase substrate $S_{1}$ and converts the substrate into fluid-phase enzyme $E_{1}$, which roughly represents fXa. Both $S_{1}$ and $E_{1}$ can bind to *P*_1_ receptors on the surfaces of activated platelets to form the platelet-bound species $S_{1}^{b}$ and $E_{1}^{b}$, respectively. Similarly, fluid-phase substrate $S_{2}$ and enzyme $E_{2}$ can bind to *P*_2_ receptors on activated platelets to form the platelet-bound species $S_{2}^{b}$ and $E_{2}^{b}$. $S_{2}$ corresponds roughly to prothrombin and $E_{1}^{b}$ is analogous to prothrombinase in that $S_{2}^{b}$ and $E_{1}^{b}$ can bind to form complex $C_{1}^{b}$ that then activates the $S_{2}^{b}$ in that complex to $E_{2}^{b}$. $E_{2}^{b}$ can then bind with $S_{1}^{b}$ to form the $C_{2}^{b}$ complex which converts its $S_{1}^{b}$ into enzyme $E_{1}^{b}$. The feedback activation of platelet-bound $S_{1}^{b}$ by $E_{2}^{b}$ is a simplified version of the feedback activity of real thrombin in increasing the concentration of the platelet-bound tenase complex which produces fXa on activated platelet surfaces. Both enzymes $E_{1}^{b}$ and $E_{2}^{b}$ can unbind from platelets becoming fluid-phase $E_{1}$ and $E_{2}$, respectively, and can then bind to other platelets. Fluid-phase $E_{2}$ is inhibited by antithrombin (AT) through a first-order reaction. Importantly, it can also react with fibrinogen in the fluid to produce fibrin monomers, as described below.

The concentrations of the fluid-phase species $S_{1}$, $E_{1}$, $S_{2}$, and $E_{2}$ evolve according to partial differential equations that describe their transport by advection and diffusion. Boundary conditions for these species couple their motion with their binding to and unbinding from surfaces. The surface densities of the platelet-bound species $S_{1}^{b}$, $E_{1}^{b}$, $S_{2}^{b}$, and $E_{2}^{b}$ evolve according to ordinary differential equations at each point on each activated platelet’s surface. These ordinary differential equations reflect the effect of the reactions that influence the rate of change of the surface densities. We ignore the possibility of diffusive transport on the platelet surfaces.

The complete set of differential equations of the reduced coagulation model is given in S1 Text. Here, we use two of those equations and the corresponding boundary conditions to illustrate the form of this part of the model. The concentration of the fluid-phase substrate $S_{1}$ satisfies the advection-diffusion equation

(4) $$\frac{\partial S_{1}}{\partial t}=-\nabla \cdot (uS_{1}-D\nabla S_{1}).$$

Because $S_{1}$ can bind to the SE-bound enzyme $E_{0}$ and to platelet-surface receptors, *P*_1_, $S_{1}$ satisfies boundary conditions both on the SE and on the surfaces of platelets. The boundary conditions are obtained by equating the diffusive flux of $S_{1}$ to a point on the boundary with the net rate of binding of $S_{1}$ to receptors on that surface:

(5) ∣−Dn^⋅∇S1∣∂Ωinj=−kC0+S1E0b+kC0−C0b,

at all points of the subendothelium and

(6) ∣−Dn^⋅∇S1∣∂Ωpla=−kS1+{N1−(S1b+E1b+C1b+C2b)}S1+kS1−S1b

at all points of each activated platelet surface. In this equation, the quantity $N_{1}-\left(S_{1}^{b}+E_{1}^{b}+C_{1}+C_{2}\right)$ is the surface density of *unoccupied P*_1_ binding sites for $S_{1}$ and $E_{1}$ on the platelet’s surface. The concentration $S_{1}$ satisfies zero diffusive-flux boundary conditions at points of the uninjured vessel walls, is set to its bulk plasma concentration at points on the inlet to the computational domain, and satisfies a zero diffusive-flux boundary condition at points of the outlet of the domain.

The surface density of the platelet-bound substrate $S_{1}^{b}$ satisfies an ordinary differential equation at all points of each activated platelet surface

(7) $$\frac{\partial S_{1}^{b}}{\partial t}=k_{S_{1}}^{+}\left\{N_{1}-\left(S_{1}^{b}+E_{1}^{b}+C_{1}^{b}+C_{2}^{b}\right)\right\}S_{1}-k_{S_{1}}^{-}S_{1}^{b}-k_{C_{2}}^{+,s}S_{1}^{b}E_{2}^{b}+k_{C_{2}}^{-}C_{2}^{b}.$$

Previous models including reactions between pairs of platelet-bound species, have treated these as volumetric reactions with the platelet-bound species quantified by their effective volume concentrations [11, 22]. Similarly, experiments measuring the rates of protein interactions of platelet-bound species report “apparent” reaction rates, treating the reactants as distributed in the solution volume [46, 47, 48]. In the current model, platelet-bound species are found on the surfaces of discrete platelets and are quantified as surface densities (amount per area). This necessitates our converting the apparent volumetric rate constants, e.g., $k_{C_{2}}^{+,v}$ into appropriate equivalents, $k_{C_{2}}^{+,s}$, for surface reactions between pairs of surface-bound species. To do this we require that the total volumetric rate of reaction for a representative volume matches the total rate of surface-bound reactions for all the platelet surfaces in that volume. See S2 Text for details.

### Fibrin polymerization model

The model of fibrin polymerization used in this paper is inspired by that in [35] and describes reactions in which fibrin monomers can polymerize to form branched fibrin oligomers. We view a fibrin monomer as a linear molecule with two half-monomer domains, and each half-monomer domain is a free reaction site; that is, each fibrin monomer has two free reaction sites. Each free site can participate in two types of reactions: linear polymerization in which two free sites come together to form a linear link, and branch formation in which three free sites come together to form a trimolecular “branch”. Branch formation produces species with more than two reaction sites and sufficient branch formation is required for gelation to occur [36]. Each fibrin oligomer, formed by these two reactions, can be described by two indices, m and b, where b is the number of branches and m+2b is the total number of monomers in the oligomer. Therefore, a free (unbound) fibrin monomer is denoted $C_{10}$ and an oligomer with m+2b monomers and b branches is denoted as $C_{mb}$. With this notation, the formation of a linear link can be described as:

(8) $$C_{m_{1},b_{1}}+C_{m_{2},b_{2}}\overset{\;k_{l}}{\to }C_{m_{1}+m_{2},b_{1}+b_{2}},$$

and the formation of a branch can be written as

(9) $$C_{m_{1},b_{1}}+C_{m_{2},b_{2}}+C_{m_{3},b_{3}}\overset{\;k_{b}}{\to }C_{m_{1}+m_{2}+m_{3}-2,b_{1}+b_{2}+b_{3}+1}.$$

The concentration of oligomers of type $C_{mb}$ is denoted $c_{mb}(x,t)$ and its time rate of change is given in Equation (69) in S3 Text. Those equations account for changes in concentrations due to polymerization reactions, due to there being a source of monomer at locations at which both fibrinogen and thrombin are present, and due to transport. For simplicity, we assume that only fibrin monomers move by advection with the fluid and by diffusion; larger oligomers are regarded as being stationary. More details and justification for this assumption can be found in the Discussion. The model also tracks the concentration of fibrinogen, denoted G(x,t) and its conversion to fibrin monomers by the enzyme thrombin ($E_{2}(x,t)$) according to Michaelis–Menten kinetics. To the extent possible, we use parameter values taken from the experimental literature (see Table 1). There is no value in the literature for the branching rate parameter $k_{b}$, and we have used experimental data [40] to estimate this parameter (see details in S3 Fig). As in [35], we exclude self-interactions between reaction sites on an oligomer so no cycles may form. Consequently, the number of free reaction sites on a $C_{mb}$ oligomer is b+2. We assume that the rates of reactions among oligomers as included in Equation (69) are proportional to the number of free reaction sites on each of the reacting species.

We are interested in the spatial-temporal dynamics of several quantities that can be defined in terms of the set of oligomer concentrations. These include the total concentration of free reaction sites R(x,t), the total concentrations of monomers in all oligomers θ(x,t), the total concentration of branches B(x,t), and the average number of monomers in an oligomer A(x,t) which we also refer to as the average cluster size. The definitions of these quantities are

(10) $$R(x,t)=\sum_{m,b}(b+2)c_{mb}(x,t),$$

(11) $$\theta (x,t)=\sum_{m,b}(m+2b)c_{mb}(x,t),$$

(12) $$B(x,t)=\sum_{m,b}bc_{mb}(x,t),$$

and

(13) $$A(x,t)=\frac{1}{\theta }\sum_{m,b}{\left(m+2b\right)}^{2}c_{mb}(x,t).$$

Following the arguments in [35, 36, 49], we define the onset of gelation at location x as the time, $t_{gel}(x)$ at which the A(x,t)→∞. Loosely speaking, gelation occurs when the average cluster size becomes infinite. As defined, we need to know all the concentrations $c_{mb}$ to calculate the quantities of interest. The differential equations for these concentrations form a doubly-infinite set precluding their solution. Instead, using a moment-generating function approach [36, 49], we derive from Equation (69) in S1 Text a small set of differential equations for select zeroth, first, and second moments of the oligomer distribution, and we use the fact that the quantities of interest defined above can be expressed as linear combinations of these moments, to derive a small closed set of differential equations directly for those quantities. Rather than tracking A(x,t), it is convenient to track $Y(x,t)=M_{02}(x,t)ˇR(x,t)$ which becomes infinite at a specific time if and only if A(x,t) becomes infinite at that time [49]. The quantity $M_{02}=\sum_{m,b}(b+2)(b+1)c_{mb}$ is a second moment of the oligomer distributions with respect to the number of branches b. The differential equations for R, θ, B, and Y are

(14) $$\frac{\partial R(x,t)}{\partial t}\;=\;-k_{l}{R(x,t)}^{2}-\frac{k_{b}}{2}R{\left(x,t\right)}^{3}\;\;+2\left(-u\cdot \nabla c_{10}(x,t)+D\Delta c_{10}(x,t)+k_{\text{cat}}E_{2}(x,t)\frac{G(x,t)}{K_{m}+G(x,t)}\right),$$

(15) $$\frac{\partial B(x,t)}{\partial t}\;=\;\frac{k_{b}}{6}{R(x,t)}^{3},$$

(16) $$\frac{\partial \theta (x,t)}{\partial t}=-u\cdot \nabla c_{10}(x,t)+D\Delta c_{10}(x,t)+k_{\text{cat}}E_{2}(x,t)\frac{G(x,t)}{K_{m}+G(x,t)},$$

(17) $$\frac{\partial Y(x,t)}{\partial t}\;=\;k_{l}Y{\left(x,t\right)}^{2}+k_{b}R(x,t)\left(\frac{{R(x,t)}^{2}}{2}+R(x,t)Y(x,t)+{Y(x,t)}^{2}\right).$$

These equations and differential equations for the fibrinogen concentration G and free fibrin monomer concentration $c_{10}$ constitute the fibrin polymerization part of our model. The former is

(18) $$\frac{\partial G(x,t)}{\partial t}\;=\;-u\cdot \nabla G(x,t)+D\Delta G(x,t)-k_{\text{cat}}E_{2}(x,t)\frac{G(x,t)}{K_{m}+G(x,t)},$$

and the latter is given in Equation (20) below. Note that the transport terms in Equations (14) and (16) describe monomer transport. By simple changes of variables, we can remove monomer transport terms from all but Equation (20) (see S3 Text for details).

It is important that Equations (14)–(17) are valid at location x only until the time at which a gel forms there. We therefore need to detect when this occurs, and to specify how concentrations evolve post gelation. Towards this end, we introduce a gelation indication function I(x,t) defined to equal 1 if a gel has formed at location x by time t, and to equal 0 otherwise. In preliminary computational experiments, we found that we could define a reasonable gelation threshold, namely gelation occurs at x at the first time $t_{gel}(x)$ at which Y(x,t) exceeds $Y_{\max}=5000$ nM. Hence, I(x,t) is defined by

(19) I(x,t)={0ift<tgel(x)1ift≥tgel(x)}.

Once gelation has occurred at x, we assume that all polymerization reactions cease there, and that the only reactions that can occur there are diffusive and advective transport of fibrin monomers and the conversion of fibrinogen to fibrin monomers by thrombin. All reactions continue at locations where a gel has not yet formed. With these assumptions, the equation for $c_{10}(x,t)$ can be written

(20) $$\frac{\partial c_{10}(x,t)}{\partial t}\;=\;-u\cdot \nabla c_{10}(x,t)+D\Delta c_{10}(x,t)+k_{\text{cat}}E_{2}(x,t)\frac{G(x,t)}{K_{m}+G(x,t)}\;\;-\delta_{I(x,t),0}\left(2k_{l}R(x,t)+k_{b}R^{2}(x,t)\right)c_{10}(x,t),$$

where $\delta_{i,j}$ is the Kronecker delta.

The fibrinogen concentration at the inlet to the domain is set to its plasma value and is assumed to satisfy zero diffusive flux boundary conditions at all points of the vessel wall and the outlet. The concentrations of all fibrin species are assumed to satisfy zero diffusive flux boundary conditions at all points of the domain boundary.

### Simulations

To simulate the dynamics of Equations (1) and (2), we utilized ANSYS Fluent 2023R2. Pressure–velocity coupling employed the coupled algorithm option; pressure was discretized with a second-order scheme, and time integration used the second-order implicit formulation option. For fluid-phase biochemical species ($S_{1},E_{1},S_{2},E_{2},G,c_{0}$) that undergo transport, the corresponding reaction diffusion advection equation (e.g. Equation (4)) along with the appropriate boundary conditions (Equations (5)–(6)) was solved in ANSYS Fluent using the user-defined scalar framework. For transported species, Fluent assembles the convection and diffusion operators using the local velocity field together with a constant diffusivity D, with all spatial terms discretized using a second-order upwind scheme and gradients evaluated with the least-squares cell-based method. Fluid-phase species that are not transported (R,B,θ,Y) were assigned zero diffusivity and convection was disabled, such that their evolution reduces to spatially local reaction ODEs, which Fluent solves implicitly. The concentrations of surface-bound species satisfy ODEs (e.g., Equation (7)) at each point of the appropriate surface, and were treated through a boundary ODE system, implemented with a user-defined function. At each time step, the procedure retrieves the current boundary values, evaluates the reaction terms, and performs an explicit forward-Euler update using the solver’s time-step size. This ensures consistent coupling between surface reactions and bulk coagulation dynamics throughout the transient simulation. Discussion of the mesh and time-step independence of the numerical solutions appears in S4 Text, S1 Fig, and S2 Fig. The simulations are performed for a physical time of one or two minutes.

## 4 Results

In this section, we show how varying the platelet packing density impacts the dynamics of both the coagulation and fibrin polymerization models. We look at three platelet plug configurations, defined as dense, medium, and loose; details about the platelet plug geometry are given in Table 2. Since the intraplug microstructure impacts transport within and around the region of the platelet plug, we also investigate how the formation of a fibrin gel is impacted by different shear conditions. All parameter values and initial conditions used for simulating the reduced coagulation model and the fibrin polymerization model are shown in Tables 1 and 3.

### Increased platelet packing density decreases fluid velocity within platelet plug

Figure 2 shows how the fluid velocity is impacted by the presence of a platelet plug and how the velocity within the platelet microstructure is modified for different packing densities. For all three configurations in Figures 2A,C,E, the maximum velocity magnitude in the center of the vessel is 18 mm/s and just upstream from the platelet aggregate and about 1 platelet diameter from the vessel wall, velocity magnitudes are around 200 μm/s. Figures 2B,D,F show how the platelet packing density influences the velocity inside the platelet plug interior. The velocities within the plugs range between 2 and 50 μm/s for the loose plug, and between 0.01 and 0.1 μm/s for the dense one.

### Platelet packing density impacts volume-integrated amounts of enzymes and fibrin

**Fig 3.**
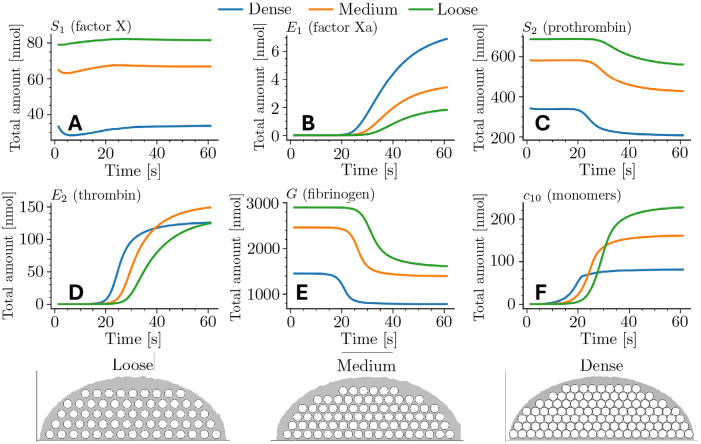
Effect of platelet-plug packing density on volume-integrated species concentrations, at a shear rate of 1000 s^−1^. Top: Time courses (0–61 s) of the total amounts of (A) $S_{1}$ (factor X), (B) $E_{1}$ (factor Xa), (C) $S_{2}$ (prothrombin), (D) $E_{2}$ (thrombin), (E) G fibrinogen, and (F) $c_{10}$ fibrin monomers for the three geometries. Bottom: Schematic half-ellipsoidal plugs with identical outer dimensions but different packing (left to right: loose, medium, dense). The grey region represents the intraplug fluid domain; all totals are computed by integrating concentrations over this region only. See Table 2 for geometric characteristics.

Figure 3 gives a spatially-integrated picture of how variations in the platelet packing density influence the consumption and production of the coagulation species and the production of fibrin monomers for shear rate 1000 s^−1^. For each fluid-phase species and time, we integrate the species’ concentration over the fluid portion of the plug, indicated in gray in the bottom row of the figure, to determine the total amount of that species within the plug. Figure 3A,C,E show the amounts of substrates $S_{1}$ and $S_{2}$ representing factor X and prothrombin, respectively, and of G representing fibrinogen for the three plug geometries. We see that the amount of each substrate decreases as the packing density increases; this is a direct consequence of the reduction of interplatelet space (54%, 37%, and 16% of the total plug area for the loose, medium, and dense plugs, see Table 2). We see in Fig. 3B,D,F that after an initial latent period of 15–30 s, the amounts of the products $E_{1}$ (factor Xa), $E_{2}$ (thrombin), and $c_{10}$ (fibrin monomer) begin to grow. The onset of the accumulation occurs earliest for the dense plug, next for the medium one, and last for the loose plug. Once started, the accumulation is fastest, intermediate, and slowest for the dense, medium, and loose plugs, respectively. For $E_{1}$, at each time the total amount is highest for the dense plug and lowest for the loose one, despite the fact that the amount of substrate $S_{1}$ runs in the opposite order. For $E_{2}$, the amount rises first and fastest for the dense plug, but the rise then slows, and by 60 s, the amounts for the medium and loose plugs have matched or exceeded that for the dense one. For $c_{10}$, the denser the plug, the sooner the rapid rise begins and the sooner it slows, so that by 60 s, there is substantially more fibrin monomer for the loose plug than for the medium or dense ones. The total amount of each of the substrates ($S_{1},S_{2},G$) approaches a quasi-steady value consistent with their consumption being balanced by replenishment due to transport.

### Location of fibrin gel initiation depends on platelet packing density and shear rate

**Fig 4.**
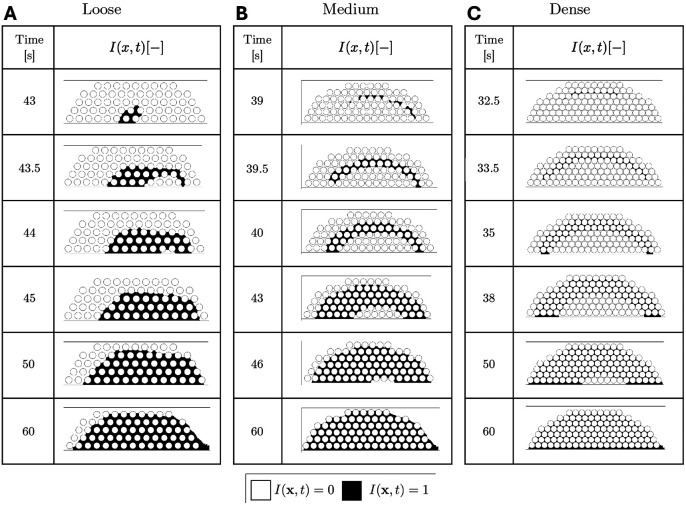
Comparison of gelation indicator I(x,t) between (A) loose, (B) medium, and (C) dense platelet plugs, at shear rate = 1000 s^−1^. Black regions in the plot correspond to spatial locations where the gelation indicator variable I(x,t)=1. Note the difference in time across different platelet configurations.

Thrombin produced by the reduced coagulation model converts fibrinogen into fibrin monomers, which polymerize and eventually form an insoluble gel. As described in the Materials and methods section, we use a gelation indicator variable, I(x,t), to track the spatial and temporal dynamics of the gel. A value I(x,t) indicates that fibrin has formed a gel at location x prior to time t. Figure 4 shows how the location of the start and subsequent development of fibrin gelation (black, corresponding to indication variable I(x,t)=1) is impacted by the platelet-packing configuration for shear rate 1000 s^−1^ up to 60 seconds. Fibrin gel formation starts earliest for the dense plug in Figure 4C, followed by the medium plug in Figure 4B, and then the loose plug in Figure 4A. Gelation starts at the bottom of the plug near the subendothelium in the loose configuration, but starts at the periphery for the medium and dense plugs. Over time, the gel front moves toward the periphery of the plug for the loose configuration. In contrast, for the medium and dense plugs, the gel front moves towards the plug’s interior. In each case, once gelation begins, it spreads quickly until (almost) the whole platelet plug is covered. Finally, we see one effect of flow in that the gel location is skewed slightly downstream, with the greatest skew seen for the loose plug.

### Higher shear rate decreases fibrin area accumulation

**Fig 5.**
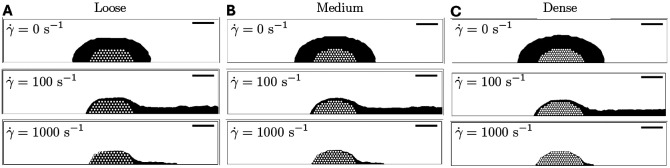
Fibrin gel area after 120 s of simulation time for (A) the loose, (B) medium, and (C) dense platelet plugs. Each subfigure corresponds to a different shear condition where (top) no shear, (middle) medium shear, $\dot{\gamma }=100\;s^{-1}$, and (bottom) high shear, $\dot{\gamma }=1000\;s^{-1}$ are shown. Horizontal bar corresponds to 25 μm.

To see the effect of longer time, Figure 5 shows the fibrin gel area at 120 seconds. We find that without flow each platelet packing density yields a fibrin gel that extends symmetrically beyond the platelet plug in all directions. With nonzero shear rate, we see that the fibrin gel area after two minutes is restricted almost exclusively to downstream of the platelet plug, with more area for shear rate 100 s^−1^ than for shear rate 1000 s^−1^ for all platelet plugs. For a given flow, the gel area downstream from the platelet aggregate decreases as the packing density increases. Some fibrin gel is seen upstream and above the platelet region for shear rate 100 s^−1^, but there is almost no fibrin gel in these locations for shear rate 1000 s^−1^. In supplement S4 Fig, we show how the fibrin gel area changes over time from 30 seconds to 120 seconds. We find that the flow conditions and the platelet plug microstructure impact the time at which a gel first appears, as well as which plug yields the largest fibrin gel area over time. In particular, as shear rate increases, the total fibrin gel area decreases for all plugs. Without flow, the dense plug has the largest gel area by 120 seconds, followed by the medium and loose plugs. However, for both shear rate $\dot{\gamma }=100\;s^{-1}$ and $\dot{\gamma }=1000\;s^{-1}$, we find that fibrin gel area at 120 seconds instead decreases as platelet packing density increases, with the loose plug resulting in the largest fibrin gel area.

### Increased platelet packing density results in a decrease in replenishment of precursor coagulation species

The previous section investigates how varying the platelet packing density impacts the overall accumulation of important coagulation and fibrin polymerization species, as well as how fibrin gel formation differs under varying flow conditions. To understand how fibrin gel formation depends on the plug microstructure, we investigate how individual species involved in coagulation and fibrin polymerization vary spatially for the different platelet packing densities.

**Fig 6.**
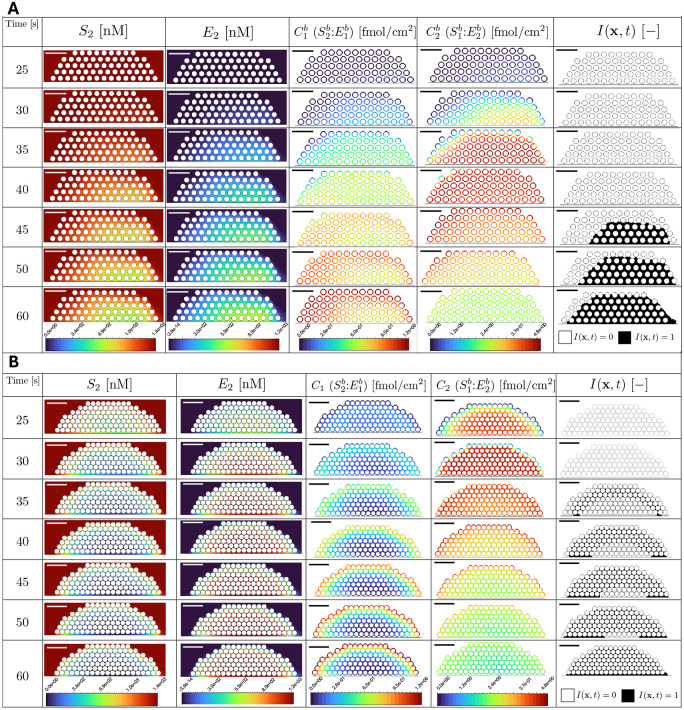
Concentrations and densities of coagulation species in the fluid and on platelet surfaces, respectively, for (A) the loose platelet plug and (B) the dense platelet plug. Each row corresponds to a different time value, the color bar for each column refers to the concentration or value of each variable, and the horizontal bar represents 10 μm.

Local concentrations influence reaction rates, which impact the formation and motion of the fibrin gel front. We show the concentrations of coagulation species, as well as the gel indicator variable, within the full two-dimensional loose and dense platelet plugs in Fig. 6 starting at time 25 s. For the loose plug (Fig. 6A), we see that the platelet-bound enzyme complex $C_{2}^{b}(S_{1}^{b}\;:E_{2}^{b})$ accumulates first on the platelet surfaces in the near-wall downstream portion of the plug. Since the catalytic rate constant for the formation of $E_{2}$ from $C_{1}^{b}$ is more than an order of magnitude larger than that for the formation of $E_{1}$ from $C_{2}^{b}$, (see Table 3), $C_{2}^{b}$ can accumulate on the surface. The $C_{2}^{b}$ complex then generates additional $E_{1}$ in the fluid (not shown) which then forms more of the platelet-bound complex $C_{1}^{b}(S_{2}^{b}\;:E_{1}^{b})$, which, in turn, produces more $E_{2}$. The accumulation of $E_{2}$ is greater in the near-wall region and is biased towards the downstream portion of the plug.

The concentrations of coagulation species for the dense plug results (Figure 6B) are markedly different from these loose plug results. This is due to the early accumulation of $E_{2}$ and an early decrease of $S_{2}$ in the interior of the dense plug indicating that thrombin production occurs earlier than for the loose plug. The earlier production of thrombin corresponds to an earlier accumulation of $C_{1}^{b}$ (the complex responsible for the production of $E_{2}$) on the surface of the platelets within the center of the domain. Additionally, the thrombin and prothrombin concentrations within the dense platelet plug remain high and low, respectively, throughout the simulation, indicating that enzyme removal and substrate replenishment by transport are small; this is not the case for the loose plug.

### Temporal dynamics of fibrin polymerization species depend on spatial location and platelet packing density

**Fig 7.**
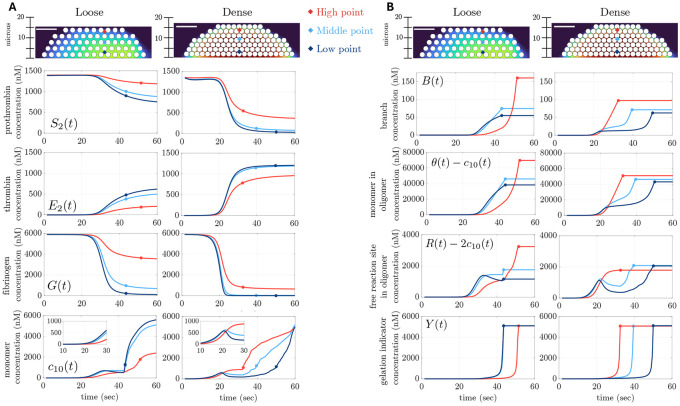
Concentrations over time of (A) coagulation species, and fibrin polymerization species, and (B) structural quantities for the (left column) loose and the (right column) dense platelet plugs. Line color refers to three spatial locations within the platelet plug: (red) high, (light blue) middle, (dark blue) low. Each dot corresponds to the gel time at that spatial location. Horizontal bar corresponds to 10 μm.

We next seek to understand how the nonuniform spatial accumulation of thrombin impacts fibrin polymerization. To allow examination of the time-courses at different spatial locations with more temporal resolution than is possible using the spatial plots of Figure 6, we choose three spatial locations (high, middle, low) within the loose and dense platelet plugs that indicates the center of the region in which thrombin accumulates within the aggregate. In Figure 7, we look at how various important concentrations change over time at these points. On each curve in this figure, a circle indicates the time of gelation at the corresponding location.

In Figure 7A, we see that at all three locations the precursor species prothrombin ($S_{2}$) and fibrinogen (G) have higher concentrations for the loose plug than for the dense one. In fact, for the dense plug, these species are greatly depleted early in the simulation (at ~ 20 s) at the lower and middle points. For prothrombin, this is due to a combination of the higher concentration of platelet reaction sites in the dense plug to which prothrombin can bind and due to the lower effectiveness of transport in replenishing it. For fibrinogen, it is due both to the high concentrations of thrombin ($E_{2}$) that develops early within the dense clot, as thrombin produced on the platelets is released into the narrow gaps between platelets, and due to the low effectiveness of transport in replenishing fibrinogen. Because less thrombin is produced near each location in the loose plug, less prothrombin is consumed and less fibrinogen is converted to fibrin monomers, so prothrombin and fibrinogen are not depleted as quickly as in the dense plug. In addition, replenishment by transport is more effective for the loose plug, especially at the top and middle points.

The early rise of the thrombin $E_{2}$ concentration at all three locations in the dense plug triggers a concurrent drop in fibrinogen and rise in the fibrin monomer concentration, $c_{10}$ (seen in Figure 7 inset). For the loose plug, a similar rise occurs but is initially limited to the lower two locations where $E_{2}$ is substantially higher than at the high location. For the dense plug, the rise in $c_{10}$ at the lower two locations is short-lived and $c_{10}$ declines shortly later because of the rapid depletion of fibrinogen there. The fibrin monomer concentration continues to rise albeit slowly at the top location in the dense plug. In the loose plug, $c_{10}$ plateaus at the lower locations and continues a slow rise at the top. Later in the simulations, at different times following the onset of gelation, $c_{10}$ rises more substantially at all locations in both plugs. This is a consequence of our assumption that fibrin can no longer oligomerize at a spatial location after gelation has occurred there. The rise in $c_{10}$ then can be due to one or both of local production of fibrin monomers that are not incorporated into oligomers there or to transport of $c_{10}$ from nearby locations at which gelation has already occurred. We discuss this further below. The circles in Figure 7A show that, for both the loose and dense plugs, gelation occurs first near the location of early monomer buildup; this occurs close to the injured wall (low point) for the loose plug and near the luminal edge (high point) for the dense plug.

Before gelation, fibrin monomers can oligomerize and thereby increase the concentrations of monomers in oligomers, $\theta -c_{10}$ and branches, B, ultimately leading to gelation. Fibrin polymerization species for the dense and loose plugs are shown in Figure 7B. Note that oligomerization stops at any spatial point once gelation occurs there and that the branch concentration B, the concentration of reaction sites in multimers ($R-2c_{10}$), and the concentration of monomers in multimers ($\theta -c_{10}$) at that location do not change after that. After gelation occurs at a first location, movement of monomers produced there to nearby locations leads to increases in the values of $c_{10}$ (Figure 7A) and contributes to polymerization at those locations. Values of R, B, and θ (Figure 7B) there increase and this potentially results in the spread of gelation to those adjacent areas. For the loose plug, gelation proceeds from near simultaneous occurrence at the lower and middle points to later occurrence at the high point. In contrast, for the dense plug, gelation starts and the high point and then proceeds sequentially to the middle and low points.

Interestingly, Figure 7B shows that, at gel time, the concentrations of monomers in multimers ($\theta -c_{10}$), branches B, and free reaction site concentration in multimers $R-2c_{10}$ are much higher for the high point in the loose plug than for the high point in the dense plug. In contrast, the loose and dense plugs have comparable values of these concentrations at gel time for the low and middle points. We discuss the causes of this difference below.

In the supplement, S5 Fig shows the full two-dimensional concentration fields at select time points for the simulations shown in Figure 7. We see there that flow-mediated transport biases the distribution of fibrin polymerization species either toward the upstream or downstream ends of the plug, depending on the plug density. In particular, S5 FigA shows that high concentrations of $R-2C_{10}$, $\theta -c_{10}$, and B are seen at the downstream (right-most) edge of the loose platelet plug, while S5 FigB shows that high concentrations of these variables are seen at the upstream (left-most) edge of the dense plug.

In this section, we have focused on results for shear rate 1000 s^−1^ . In supplemental figures S7 Fig, S8 Fig, and S9 Fig, we compare results for selected spatial locations in the loose and dense plugs obtained in simulations with shear rate 1000 s^−1^ with results obtained in simulations without flow. Those figures show concentrations of coagulation species and fibrin polymerization species along with the rates of fibrinogen transport, fibrinogen conversion to fibrin monomers, monomer transport, and monomer incorporation into larger oligomers. In brief, rates of fibrin monomer supply and consumption and the fibrin species concentrations are smaller for the no flow case for both plug densities. Coagulation species concentrations for the dense plug are largely the same with and without flow. For the loose plug, enzymatic species accumulate more without flow because of a smaller degree of washout. The difference in the fibrin species concentrations and monomer supply and consumption rates between the flow and no-flow cases is larger for the loose plug than for the dense one. These differences in polymerization dynamics result in differences in the gelation time. For the loose plug without flow, gelation does not occur at any of the three spatial locations within the first 60 seconds. For the dense plug without flow, gelation occurs within 60 seconds at the top and middle point but not at the bottom point while it occurs at all three locations for the dense plug with flow. In the next section we discuss how fibrin monomer production and transport influence where gelation begins and how the gel region expands for the loose and dense plugs.

### Fibrin monomer concentration and transport influence gelation location and motion

**Fig 8.**
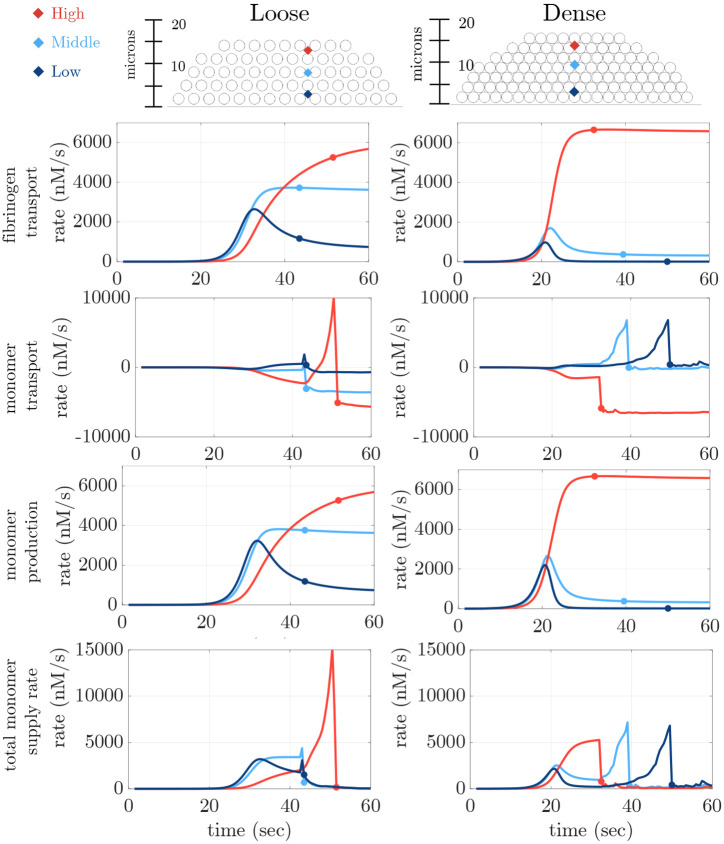
Rates over time (from top to bottom row) of fibrinogen transport (−u⋅∇G+DΔG)), monomer transport ($-u\cdot \nabla c_{10}+D\Delta c_{10}$), monomer production ($k_{\text{cat}}\frac{E_{2}G}{K_{m}+G}$) and total monomer supply rate ($k_{\text{cat}}\frac{E_{2}G}{K_{m}+G}+-u\cdot \nabla c_{10}+D\Delta c_{10}$). Line color refers to three spatial locations within the platelet plug: (red) high, (light blue) middle, (dark blue) low. Each dot corresponds to the gel time at that spatial location.

The results in Figures 6 and 7 indicate that the spatial location of the emergence of a gel depends on the platelet packing density. In particular, the regions within either the dense or loose platelet where $c_{10}$ accumulates early correlate with where the gelation indicator I first becomes nonzero. In this section, we investigate the mechanisms by which $c_{10}$ is produced in a given spatial region. Figure 8 shows the impact of flow and platelet packing density on the rate of transport of fibrinogen, −u⋅∇G+DΔG, and fibrin monomers, $-u\cdot \nabla c_{10}+D\Delta c_{10}$, as well as the rate of production of monomer and the total monomer supply rate. We define the fibrin monomer production rate as $k_{\text{cat}}E_{2}\frac{G}{K_{m}+G}$ and the total monomer supply rate as the sum of the rates of monomer transport and monomer production.

In Fig. 8, positive values indicate transport to and negative values indicate transport away from the location. Here, we define transport as both convective and diffusive transport. In both the dense and loose plugs, the rate of fibrinogen transport is almost identical to the rate of monomer production. Since $c_{10}$ production is dependent on G and the only source of G is from transport in the fluid, this indicates that the production of fibrin monomer is limited by the transport of fibrinogen. Within the interior of the platelet plug (at the mid and low point), the maximum rate of transport of fibrinogen for the dense plug is almost an order of magnitude smaller than its maximum rate of transport in the interior of the loose plug, where fibrinogen transport is sustained. This difference is largest at the low point, where transport of fibrinogen for the dense plug drops quickly to almost zero compared to the sustained fibrinogen transport seen in the loose case.

The differences in fibrinogen transport strongly impact the monomer production rates for the different density plugs. While monomer production is sustained near the periphery of both plugs, at the lower points in the interior of the dense plug it continues at a low level, despite high thrombin concentrations. This is due to fibrinogen depletion, and the rate of fibrinogen resupply by transport is low. For the loose plug, monomer production at the lower points continues at a rate more than twice that for the dense plug because transport-mediated delivery of fibrinogen to these locations persists. Based on the Peclet number in the dense plug (see Table 2), transport in this case is primarily due to diffusion, while the Peclet number in the loose plug indicates diffusive and advective transport are both important.

For both plugs, fibrin monomers leave the peripheral region near the high point (the transport rate there is negative) escaping into the bulk fluid and moving toward the plug’s interior (the transport rates there are slightly positive). At the high point in the dense plug and at the mid points for the loose plug, monomer transport becomes substantially more negative once gelation occurs at those points, because monomers newly produced there are not incorporated into the oligomers after gelation. As a consequence, we see that at the mid point in the dense plug, monomer transport becomes positive following gelation at the top point; similarly monomer transport becomes positive at the top point in the loose plug following gelation at the lower points in that plug.

The overall rate of supply of fibrin monomer at a specific location is the sum of the monomer production rate and monomer transport rates there. We see that gelation occurs earliest at the location with the greatest sustained early total rate of monomer supply. For the loose plug, gelation occurs earliest and almost simultaneously at the mid and low points. For the dense plug, it occurs first at the high point. First gelation happens about 10 seconds sooner for the dense plug than for the loose one, a consequence mainly of the high rate of monomer production near the periphery of the dense plug because of the simultaneous presence of high thrombin and fibrinogen concentrations. At the lower points in the dense plug, the monomer supply rate initially rises after thrombin is produced but quickly drops because of depletion of fibrinogen. Progress towards gelation at these points is slow with a gradual increase in branch concentration B and oligomeric mass $\theta -c_{10}$ as shown in Figure 7B. But this progress becomes more rapid after gelation occurs at the top point because of transport of monomers to these points from the plug periphery.

The movement of the gel front is further illustrated in Supplement S6 Fig which shows transport and production rates in two dimensions. In particular, we can clearly see the gel front there moving toward the periphery (from low to high) in the loose case and towards the injured wall (from high to low) in the dense case.

The timing of the later gelation at the top point of the loose plug and the bottom points of the dense plug is influenced by monomer transport from other points in each plug at which a gel has already formed. The magnitude of this transport is, in turn, affected by our modeling assumption that after gelation occurs at a particular spatial location, further incorporation of monomers into oligomers at that location ceases. Recall that some assumption about post-gelation reactions is needed because the differential equations for B, θ, and R are not valid after gel time. We sought to obtain an estimate of the magnitude of the effect of this assumption on the timing of the subsequent gelation events. To do this, we modified a version of our non-spatial fibrin branching model [35] that incorporates as inputs the thrombin and fibrinogen concentration time courses from a particular location in our current simulations, and to produce fibrin monomers through the action of the input thrombin on the input fibrinogen. The resulting fibrin monomer concentration is therefore unaffected by fibrin transport from other locations. Supplement S10 Fig shows the gel times and time courses of $c_{10}$ and R when this is done for the low point in the dense plug. We see that excess transport from other locations in the current, two-dimensional model increases the speed of gelation by only a few seconds, compared to the non-spatial fibrin branching model.

## 5 Discussion

The formation of a thrombus involves the interplay of platelet activation and aggregation, the production of thrombin through the coagulation reactions, and the subsequent formation of a fibrin mesh that stabilizes the growing platelet aggregate under flow conditions. Here, we present a mathematical framework that examines how physical characteristics of the platelet microstructure can impact the production of thrombin and fibrin polymerization. Our framework incorporates a reduced model of coagulation reactions, most of which occurs on the surfaces of platelets in a preformed plug or aggregate made up of discrete platelets. The framework also includes a model of fibrin polymerization which allows for a branched fibrin mesh to form thus making gelation possible. Under flow, blood moves between the stationary platelets in the aggregate and therefore coagulation proteins and fibrin polymerization species can move within the aggregate by advection and diffusion. To our knowledge, this is the first model where coagulation reactions are modeled on discrete platelet surfaces and subsequent fibrin polymerization in the surrounding space is tracked.

By varying both the spacing between platelets in the plug and the flow conditions, we studied how they, along with coagulation reactions on discrete platelet surfaces, impact the concentrations of fluid-phase coagulation species, in particular thrombin, in the adjacent fluid, and how the thrombin concentration influences fibrin polymerization. In our simulations, gelation occured first at the periphery of the medium and dense plugs, whereas it occurred first near the vessel wall for the loose plug. This spatial shift can be understood from the interplay between platelet plug density, thrombin generation, and fibrinogen transport. Dense plugs provide more platelet surface area but less fluid space between platelets, promoting rapid thrombin formation and leading to high local thrombin concentrations at some locations. Some concentrations of thrombin in our simulations are high compared to values reported in some studies of pure continuum models [11, 21, 22, 34] in which thrombin is distributed in the entire volume occupied by fluid and platelets. In our current work, the thrombin released from platelet surfaces into the fluid is confined to the fluid region between the platelets. Some microfluidic experiments and computational results suggest that thrombin concentrations can be as high as 1000 nM within the thrombus core [50, 51]; however, the majority of thrombin produced there is likely bound to fibrin fibers, which our model does not account for.

The high concentration of thrombin in the dense plug impacts fibrin polymerization dynamics through the acceleration of fibrinogen consumption and monomer production. However, because fluid velocities in the dense plug are extremely low, fibrinogen replenishment is effective mainly at the plug periphery. As a result, after a short initial period, monomer production, and subsequently branch and oligomer formation in the plug interior is very slow. Branch and oligomer formation are sustained only near the outer regions of the plug, causing gelation to start there and to propagate inward. Some fibrinogen may be resupplied to the near-wall region through a diffusive-dominated mechanism, but it is quickly converted by high thrombin found within the plug interior. Similar results are seen in [30], where transport is due mainly to diffusion and increasing the shear rate has little impact on velocity within the platelet aggregate. In contrast, for the loose plug we see noticeably greater fibrinogen transport into the plug interior, especially at early time points. Although the lower platelet surface area leads to slower production of coagulation enzymes compared to that in the dense plug, the more effective replenishment of fibrinogen supports persistent monomer generation deeper within the plug. Consequently, gelation begins deep in the plug interior for the loose plug. It is interesting that despite producing roughly twice as much monomer overall as the dense plug (Figure 3), the loose plug gels initially at a later time point and in a different spatial pattern than for the denser plugs, most likely due to washout in the loose plug.

As the plug becomes denser, our simulations show increasingly hindered intraplug transport of fibrinogen, causing fibrin formation to proceed sooner and faster near the plug’s lumenal edge. This is consistent with findings of Stalker et al., who observed limited plasma-protein accessibility to the dense platelet-core and observed a band-like zone around the dense core where plasma proteins could still penetrate [4]. Other in vivo studies have shown that after vascular injury the early thrombus surface is platelet-rich, but as the thrombus stabilizes and contracts it becomes progressively covered by a fibrin network interspersed with platelet clusters [13, 28]. Taken together, these observations suggest that our loose-plug configuration may represent an initial platelet plug, where fibrin can form at the base, providing the network required for subsequent clot contraction [52]. Conversely, the dense-plug configuration may resemble a clot approaching a stabilized, contracted state, in which fibrin formation becomes largely confined to the outer surface of the compact platelet core. This interpretation is further supported by Mirramezani et al. (2018), who analyzed stabilized plasma clots formed over 10 minutes and reported interplatelet gap sizes ranging from 0.06 to 0.6 μm [53]. The gap sizes in our dense and medium plug models (0.1 μm and 0.5 μm, respectively) fall within this experimentally observed range, whereas the loose plug gap size (1.0 μm) lies outside of it, consistent with a plug that has not yet undergone contraction or stabilization. These observations suggest an explanation for why contraction occurs relatively late: an early, strongly compact platelet plug would seal the injury more rapidly, but would also become too compact to permit the fibrinogen transport and intraplug fibrin formation, which is necessary for robust thrombus stabilization [52].

Furthermore, our simulations showed that with increasing shear rate, the total volume of gel decreased. This trend is consistent with experimental observations under flow. Swieringa et al. reported that at a relatively low venous shear rate (150 s^−1^), the heights of platelet plugs were low but the plugs were surrounded by an extensive fibrin network that extended far outside the platelet aggregates, whereas at higher shear rates, fibrin outgrowth was more restricted [9]. Likewise, Onasoga et al. demonstrated on TF–rich microspots that increasing the shear rate from 50 to 1000 s^−1^ led to a significant reduction in fibrin gel height [54]. In line with these studies, our results demonstrate that at higher shear the convective washout of thrombin and fibrin(ogen) factors limits the development of an extended “fibrin tail" downstream of the plug.

Lastly, in our simulations fibrin gel formation was initiated within one minute. This is consistent with in vivo imaging studies, which show that fibrin accumulation can begin within one minute after laser-induced vascular injury in mice [4, 10, 55]. In contrast, many in vitro microfluidic assays exhibit longer lag times of typically several minutes before detectable fibrin formation, depending on the coagulant surface and shear rate [9, 54, 56]. Thus, the relatively early onset of fibrin formation in our model aligns more closely with in vivo kinetics and likely reflects a stronger local procoagulant drive than in typical in vitro systems. However, we cannot directly compare our model to the in vivo situation since our model is still a simplified model that does not fully capture thrombosis. It has also been shown in static conditions that increasing thrombin concentration results in a “finer" fibrin gel with a higher branch point density [40], and spatial variation in thrombin concentration can result in a heterogeneous fibrin gel [57, 58]. While we use parameter values that are determined from measurements in static experiments [40, 41, 42], we do not qualitatively recapitulate results in static conditions. In the situation where transport can have a strong effect and where platelet membrane surfaces can make an impact, our results suggest that a different outcome can be observed.

While our model incorporates many essential components of the blood clotting process, it does have limitations. We have implemented a reduced model of thrombin generation which accounts for the fact that thrombin production occurs on surfaces of activated platelets in the thrombus. Our main interest is capturing platelet surface-mediated thrombin generation, with thrombin produced to an extent and on time scales similar to those seen with a full model of coagulation [21]. A more comprehensive description of coagulation can contain more than 130 differential equations and incorporates inhibitory pathways and multiple feedback loops [22, 59]. Such a model can be used to for a more precise dissection of the coagulation system in the presence of platelets and flow [11, 60].

There is no known kinetic parameter for fibrin branching, so we have estimated the branching parameter, $k_{b}$, for the fibrin polymerization model with experimental data from [40]. In our model, gel times and in turn the gel structure are moderately sensitive to this parameter. Incorporating more experimental data and using parameter estimation techniques to better quantify the branching rate remains future work. We also make simplifying assumptions on the transport of oligomers and monomers. In order to derive the small set of ODEs for the branch point, reaction site, and total monomer in oligomer densities in the kinetic polymerization framework, one can either assume all oligomers diffuse with the same diffusion coefficient or a finitely-many can have a non-zero diffusion coefficient. Here, we assume that only fibrinogen and fibrin monomers can diffuse and advect in the fluid, not larger oligomers. It is not difficult to extend transport to include a finite number of small oligomers as in [36], but we believe our current assumption is reasonable given that our interest in understanding in vivo situations where diffusion of large oligomers may be of little significance. If a blood clot forms in the extravascular space in the presence of protein matrices and other cells, the spatial gaps are likely to be small, limiting the importance of the diffusion of large oligomers. Furthermore, there is limited room for diffusion of large oligomers in between platelets in dense plugs.

Because the differential equations for the moments of the oligomer distribution (see Supplement S3 Text) used to derive the differential equations for R, θ, and B are valid only until a gel is formed [35, 36, 49, 61], we were required to make a modeling assumption for how the dynamics at a particular spatial location ought to proceed after gelation occurs there. A model in which oligomers could contribute to post-gelation thickening of fibers is not yet available, so we made the assumption that all polymerization reactions cease at a point in space when gelation first occurs there. The consequence of this is that the only fate open to monomers produced there is to move to another location in the plug; this can alter the speed of polymerization reactions at that second location. In Supplement S10 Fig, we present evidence that the magnitude of this effect is small, by comparing gel times in the simulations reported here with ones obtained using a non-spatial model (therefore having no transport).

Our modeling framework can be used to study other important aspects of fibrin polymerization and coagulation under flow. Here, we chose to model preformed platelet plugs consisting of stationary, solid particles in a fluid domain without explicitly modeling physical connections between platelets. In reality, fibrinogen and fibrin polymers are important ligands for receptors on platelet surfaces like *α*IIb*β*3 and GPVI, which are responsible for platelet aggregation and platelet activation, respectively [52, 62]. The formation of fibrin polymer in the intraplug gaps mediated by these fibrin(ogen)-receptor interactions could also hinder flow in between platelets. Future work includes incorporating the permeability of the fibrin polymer between platelets using a Darcy term. Finally, previous computational and experimental models have demonstrated that TF surface density exhibits a distinct threshold behavior for thrombus initiation, characterized by minimal clot formation at low TF levels and rapid thrombin generation above a critical TF concentration [12, 63]. This model could investigate the impact of TF surface density and help understand the exact nature of the interaction between platelet plug microstructure, TF density, fibrin polymerization, and shear rate.

## Supplementary Material

Supplement 1

## Figures and Tables

**Table 1.** Parameters and chemical species (with their corresponding variables) in the discrete platelet model, the reduced model of coagulation, and the fibrin polymerization model. Initial concentrations are given, where species without a concentration are initialized to zero.

Parameter	Value	Reference
Diffusion coefficient (D)	5 × 10^-7^ cm^2^/s	[22]
Dynamic viscosity (μ)	1.2 mPa·s	[38]
Fluid density (ρ)	1.025 g/cm^3^	[39]
Platelet activation rate ($k_{\text{cat}}$)	0.05 s^-1^	Estimated
Platelet diameter ($P_{\text{diam}}$)	2.46 μm	[34]
Number of *P*_1_ receptors per platelet ($N_{1}^{\text{plt}}$)	2700	[34]
Number of *P*_2_ receptors per platelet ($N_{2}^{\text{plt}}$)	2000	[34]
Total *P*_1_ binding site density ($N_{1}$)	2.35 × 10^1^ fmol/cm^2^	[34],S2 Text
Total *P*_2_ binding site density ($N_{2}$)	1.74 × 10^1^ fmol/cm^2^	[34],S2 Text
Volumetric $C_{1}^{b}$ association rate ($k_{C_{1}}^{+,v}$)	1.03 × 10^8^ M^-1^ s^-1^	[34]
Volumetric $C_{2}^{b}$ association rate ($k_{C_{2}}^{+,v}$)	1.73 × 10^7^ M^-1^ s^-1^	[34]
Surface $C_{1}^{b}$ association rate ($k_{C_{1}}^{+,s}$)	1.08 cm^2^ fmol^-1^ s^-1^	Calculated in Equations (63)–(68)
Surface $C_{2}^{b}$ association rate ($k_{C_{2}}^{+,s}$)	1.82 × 10^-1^ cm^2^ fmol^-1^ s^-1^	Calculated in Equations (63)–(68)
Fibrin branching rate ($k_{b}$)	1.5 ×10^9^ M^-2^s^-1^	Estimated from [40]
Fibrin linking rate ($k_{l}$)	8.2 × 10^5^ M^-1^s^-1^	[41]
Fibrinogen conversion to fibrin monomer ($k_{\text{cat}}$)	84 s^-1^	[42]
Michaelis–Menten constant ($K_{m}$)	7.2 ×10^-6^ M	[42]
Gelation threshold value for $Y(x,t)(Y_{\max})$	5000	Estimated
Thrombin inhibition by antithrombin ($k_{AT}$)	0.0336 s^-1^	Calculated from [43, 44]
Chemical	Concentration/density	Reference
$E_{0}$ (TF:fVIIa)	0.02 fmol/cm^2^	This study
$S_{1}$ (fX)	0.17 ×10^-6^ M	[34]
$S_{2}$ (Prothrombin)	1.4 ×10^-6^ M	[34]
G (Fibrinogen)	5.88 ×10^-6^ M	[45]
$E_{1}$ (fXa)	–	–
$E_{2}$ (Thrombin)	–	–
$c_{10}$ (Free monomers)	–	–
B (Branches)	–	–
θ (Monomers in oligomers)	–	–
R (Reactive sites)	**—**	**—**
Y (Gelation indication)	–	–

**Table 2.** Platelet plug characteristics for different packing densities in predefined region.

Characteristic	Dense	Medium	Loose
Number of platelets [–]	114	79	63
Concentration [platelets/mm^3^]	6.84 × 10^7^	4.44 × 10^7^	2.78 × 10^7^
Area occupied (2D) [%]	83.89	62.86	46.02
Free-space (2D) [%]	16.12	37.14	53.98
Free-space (2D) [μm^2^]	247.12	417.98	492.81
Gap size [μm]	0.1	0.5	1.0
Average intraplug velocity [μm/s]	28.12	76.55	110.76
Average intraplug Peclet number [–]	0.056	0.77	2.22

**Table 3.** Reactions on subendothelium and platelet surface (kinetic constants in solution and on the surface), with relevant parameter and parameter values given. Surface reaction rates are calculated from volumetric reaction rates in S2 Text. All reaction rates $k_{C_{i}}^{+,s}$ are taken from [34].

Activation (of –, by –)	Complex	Product	$k_{C_{i}}^{+,s}$ (cm^2^ fmol^-1^ s^-1^)	$k_{C_{i}}^{+,v}$ (M^-1^ s^-1^)	$k_{C_{i}}^{-}$ (s^-1^)	$k_{C_{i}}^{\text{cat}}$ (s-1)
($S_{1},E_{0}^{b}$)	$C_{0}^{b}$	$E_{1}$	–	8.95 × 10^6^	1.0	1.15
($S_{2}^{b},E_{1}^{b}$)	$C_{1}^{b}$	$E_{2}^{b}$	1.08	1.03 × 10^8^	1.0	30.0
($S_{1}^{b},E_{2}^{b}$)	$C_{2}^{b}$	$E_{1}^{b}$	1.82 × 10^-1^	1.73 × 10^8^	1.0	0.23

Binding of	Reactants	Product	$k_{{*}_{i}}^{+}$ (M^-1^ s^-^	$k_{{*}_{i}}^{-}$ (s^-1^)
$S_{1}$ (X)	$S_{1},P_{1}$	$S_{1}^{b}$	5.7 × 10^7^	0.17
$E_{1}$ (Xa)	$E_{1},P_{1}$	$E_{1}^{b}$	1.0 × 10^7^	2.5 × 10^-2^
$S_{2}$ (II)	$S_{2},P_{2}$	$S_{2}^{b}$	1.0 × 10^7^	5.9
$E_{2}$ (IIa)	$E_{2},P_{2}$	$E_{2}^{b}$	1.0 × 10^7^	5.9
