# Supplementary material for: Platelet plug microstructure and flow modulate fibrin gelation dynamics: Insights from computational simulations
Source: ArXiv. 2026 Apr 9:arXiv:2604.07844v1. Preprint. [Version 1] (PMC13082137)

6 Supporting information

Supporting information

S1 Text. Reduced coagulation model details. The reduced model describing the production of thrombin uses the following enzymatic reactions, which occur on the subendothelium

$$E_0^b + S_1 \underset{k_{C_0}^-}{\overset{k_{C_0}^+}{\rightleftarrows}} C_0^b \xrightarrow{k_{C_0}^{\text{cat}}} E_0^b + E_1, \tag{21}$$

or on platelet surfaces,

$$S_2^b + E_1^b \underset{k_{C_1}^-}{\overset{k_{C_1}^{+,s}}{\rightleftarrows}} C_1^b \xrightarrow{k_{C_1}^{\text{cat}}} E_2^b + E_1^b, \tag{22}$$

$$S_1^b + E_2^b \underset{k_{C_2}^-}{\overset{k_{C_2}^{+,s}}{\rightleftarrows}} C_2^b \xrightarrow{k_{C_2}^{\text{cat}}} E_2^b + E_1^b, \tag{23}$$

$$P_1 + S_1 \underset{k_{S_1}^-}{\overset{k_{S_1}^+}{\rightleftarrows}} S_1^b, \tag{24}$$

$$P_2 + S_2 \underset{k_{S_2}^-}{\overset{k_{S_2}^+}{\rightleftarrows}} S_2^b, \tag{25}$$

$$P_1 + E_1 \underset{k_{E_1}^-}{\overset{k_{E_1}^+}{\rightleftarrows}} E_1^b, \tag{26}$$

$$P_2 + E_2 \underset{k_{E_2}^-}{\overset{k_{E_2}^+}{\rightleftarrows}} E_2^b. \tag{27}$$

Fluid-phase Species: S_1, E_1, S_2, E_2

$$\frac{\partial S_1}{\partial t} = -\nabla \cdot (\mathbf{u}S_1 - D\nabla S_1), \tag{28}$$

with boundary conditions

$$-D\,\hat{n}\cdot\nabla S_1\Big|_{\partial\Omega_{\text{inj}}} = -k_{C_0}^+ S_1 E_0^b + k_{C_0}^- C_0^b,$$

and

$$-D\,\hat{n}\cdot\nabla S_1\Big|_{\partial\Omega_{\text{pla}}} = -k_{S_1}^+ \left\{ N_1 - \left(S_1^b + E_1^b + C_1^b + C_2^b \right) \right\} S_1 + k_{S_1}^- S_1^b.$$

$$\frac{\partial S_2}{\partial t} = -\nabla \cdot (\mathbf{u}S_2 - D\nabla S_2), \tag{29}$$

with boundary condition

$$-D\,\hat{n}\cdot\nabla S_2\Big|_{\partial\Omega_{\text{pla}}} = -k_{S_2}^{+}\left\{N_2 - \left(S_2^b + E_2^b + C_1^b + C_2^b\right)\right\}S_2 + k_{S_2}^{-}S_2^b.$$

$$\frac{\partial E_1}{\partial t} = -\nabla\cdot\left(\mathbf{u}E_1 - D\nabla E_1\right), \tag{30}$$

with boundary conditions

$$-D\hat{\mathbf{n}}\cdot\nabla E_1\big|_{\partial\Omega_{\text{inj}}} = k_{C_0}^{\text{cat}}C_0^b,$$

and

$$-D\hat{\mathbf{n}}\cdot\nabla E_1\big|_{\partial\Omega_{\text{pla}}} = -k_{E_1}^{+}\left\{N_1 - \left(S_1^b + E_1^b + C_1^b + C_2^b\right)\right\}E_1 + k_{E_1}^{-}E_1^b,$$

$$\frac{\partial E_2}{\partial t} = -\nabla\cdot\left(\mathbf{u}E_2 - D\nabla E_2\right) - k_{AT}E_2, \tag{31}$$

with boundary condition

$$-D\hat{\mathbf{n}}\cdot\nabla E_2\big|_{\partial\Omega_{\text{pla}}} = -k_{E_2}^{+}\left\{N_2 - \left(S_2^b + E_2^b + C_1^b + C_2^b\right)\right\}E_2 + k_{E_2}^{-}E_2^b.$$

Subendothelium-bound Species: E_0^b, C_0^b

$$\frac{\partial E_0^b}{\partial t} = -k_{C_0}^{+}S_1E_0^b + \left(k_{C_0}^{-} + k_{C_0}^{\text{cat}}\right)C_0^b, \quad \text{on } \partial\Omega_{\text{inj}} \tag{32}$$

$$\frac{\partial C_0^b}{\partial t} = k_{C_0}^{+}S_1E_0^b - \left(k_{C_0}^{-} + k_{C_0}^{\text{cat}}\right)C_0^b, \quad \text{on } \partial\Omega_{\text{inj}} \tag{33}$$

Platelet-bound Species: S_1^b, E_1^b, S_2^b, E_2^b, C_1^b, C_2^b

$$\frac{\partial S_1^b}{\partial t} = k_{S_1}^{+}\left\{N_1 - \left(S_1^b + E_1^b + C_1^b + C_2^b\right)\right\}S_1 - k_{S_1}^{-}S_1^b - k_{C_2}^{+,s}S_1^bE_2^b + k_{C_2}^{-}C_2^b \tag{34}$$

$$\frac{\partial S_2^b}{\partial t} = k_{S_2}^{+}\left\{N_2 - \left(S_2^b + E_2^b + C_1^b + C_2^b\right)\right\}S_2 - k_{S_2}^{-}S_2^b - k_{C_1}^{+,s}S_2^bE_1^b + k_{C_1}^{-}C_1^b \tag{35}$$

$$\frac{\partial E_1^b}{\partial t} = k_{E_1}^{+}\left\{N_1 - \left(S_1^b + E_1^b + C_1^b + C_2^b\right)\right\}E_1 - k_{E_1}^{-}E_1^b \tag{36}$$

$$- k_{C_1}^{+,s}S_2^bE_1^b + \left(k_{C_1}^{-} + k_{C_1}^{\text{cat}}\right)C_1^b + k_{C_2}^{\text{cat}}C_2^b \tag{37}$$

$$\frac{\partial E_2^b}{\partial t} = k_{E_2}^{+}\left\{N_2 - \left(S_2^b + E_2^b + C_1^b + C_2^b\right)\right\}E_2 - k_{E_2}^{-}E_2^b \tag{38}$$

$$- k_{C_2}^{+,s}S_1^bE_2^b + \left(k_{C_2}^{-} + k_{C_2}^{\text{cat}}\right)C_2^b + k_{C_1}^{\text{cat}}C_1^b \tag{39}$$

$$\frac{\partial C_1^b}{\partial t} = k_{C_1}^{+,s}S_2^bE_1^b - \left(k_{C_1}^{-} + k_{C_1}^{\text{cat}}\right)C_1^b, \tag{40}$$

$$\frac{\partial C_2^b}{\partial t} = k_{C_2}^{+,s}S_1^bE_2^b - \left(k_{C_2}^{-} + k_{C_2}^{\text{cat}}\right)C_2^b \tag{41}$$

S2 Text. Conversion from volumetric to surface reaction rates. Our 2D computational framework introduces coagulation reactions on the 2D surfaces of discrete platelets, so platelet-bound species and rate constants are quantified as surface densities (amount per area). Experimental measurements and previous mathematical frameworks treated these species and reactions in the solution volume, so platelet-bound species are measured as volume concentrations and reaction rates are measured as volumetric rate constants. The following derivations show equations for both fluid-phase, volumetric species and equations for surface-bound species measured in amount per area. Here, we use variables and reaction rates with bars to denote surface-bound species, and no bars to represent volumetric species.

Derivation of platelet surface binding rates. First, we derive the rate for fluid-phase species to bind to the surfaces of platelets. As an example, we focus on the binding and unbinding of S_1 to the platelet receptor, P_1. To derive these rates, we first introduce an equation which describes all species and reaction rates as volumetric [34]. Here, all reaction rates and species are be measured in $\frac{\text{moles}}{\text{dm}^3}$.

$$-\frac{dS_1^b}{dt} = \frac{dS_1}{dt} = -k_{S_1}^+ \left(\underbrace{\frac{P^b N_1^{\text{plt}}}{N_A}}_{\substack{\text{total concentration} \\ \text{of binding sites}}} - \underbrace{\left(S_1^b + E_1^b + C_1^b + C_2^b\right)}_{\substack{\text{concentration of occupied} \\ \text{binding sites}}} \right) S_1 + k_{S_1}^- S_1^b, \tag{42}$$

where P^b is the number of bound platelets per dm^3 (liter) in a given volume, V, N_1^{plt} is the number of P_1 receptors per platelet, and N_A is 6.022×10^{23} mol^{-1} (Avogadro's constant). This implies that $\frac{dS_1}{dt} \times V$ would be $\frac{\text{moles}}{\text{sec}}$ of S_1 moving between fluid-phase and platelet-bound S_1.

We now introduce a similar equation but with reaction rates and species measured as surface densities $\left(\frac{\text{moles}}{\text{dm}^2}\right)$ and denoted with a bar, so that

$$-\frac{d\bar{S}_1^b}{dt} = -\bar{k}_{\bar{S}_1}^+ \left(\underbrace{\frac{N_1^{\text{plt}}}{N_A A_p}}_{\substack{\text{total density} \\ \text{of binding sites}}} - \underbrace{\left(\bar{S}_1^b + \bar{E}_1^b + \bar{C}_1^b + \bar{C}_2^b\right)}_{\substack{\text{density of occupied} \\ \text{binding sites}}} \right) S_1 + \bar{k}_{\bar{S}_1}^- \bar{S}_1^b, \tag{43}$$

where $A_p = 4\pi(0.5 \times P_{\text{diam}})^2$ is the characteristic platelet surface area measured in dm^2. Note that since each term on the right-hand side must have units $\frac{\text{moles}}{\text{dm}^2 \text{ sec}}$, $\bar{k}_{\bar{S}_1}^+$ must have units $\frac{1}{\frac{\text{moles}}{\text{dm}^3} \text{ sec}}$.

Suppose that in volume V there are N^b bound platelets, so

$$P^b V = N^b, \tag{44}$$

that each platelet has the same radius, and that all of these platelets see the same fluid-phase S_1. Then the rate at which the amount of S_1 moves between fluid-phase and

platelet-bound $\left(\frac{\text{moles}}{\text{sec}}\right)$ in Equation (43) is

$$-\left(N^b\,A_p\right)\frac{d\bar{S}_1^b}{dt} = -\bar{k}^+_{\bar{S}_1}N^b\,A_p\left(\frac{N_1^{\text{plt}}}{N_A\,A_p} - \left(\bar{S}_1^b + \bar{E}_1^b + \bar{C}_1^b + \bar{C}_2^b\right)\right)S_1 + \bar{k}^-_{\bar{S}_1}\left(N^b\,A_p\right)\bar{S}_1^b, \quad (45)$$

and the rate of the amount of S_1 moving between fluid-phase and platelet-bound $\left(\frac{\text{moles}}{\text{sec}}\right)$ in Equation (42) is

$$-V\frac{dS_1^b}{dt} = -k^+_{S_1}V\left(\frac{P^bN_1^{\text{plt}}}{N_A} - \left(S_1^b - E_1^b - C_1^b - C_2^b\right)\right)S_1 + k^-_{S_1}VS_1^b. \quad (46)$$

To have the same rate transfer for the unbinding of S_1^b, then

$$k^-_{S_1}\left(VS_1^b\right) = \left(N^b\,A_p\,\bar{S}_1^b\right)\bar{k}^-_{\bar{S}_1}. \quad (47)$$

Since VS_1^b and $N^bA_p\bar{S}_1^b$ both represent the moles of S_1^b in volume V, then we have that

$$k^-_{S_1} = \bar{k}^-_{\bar{S}_1}. \quad (48)$$

Similarly, to have the same net transfer rate for the binding of S_1 then

$$k^+_{S_1}V\left(\frac{P^bN_1^{\text{plt}}}{N_A} - \left(S_1^b + E_1^b + C_1^b + C_2^b\right)\right) = \bar{k}^+_{\bar{S}_1}N^b\,A_p\left(\frac{N_1^{\text{plt}}}{N_A\,A_p} - \left(\bar{S}_1^b + \bar{E}_1^b + \bar{C}_1^b + \bar{C}_2^b\right)\right). \quad (49)$$

Again, the terms multiplied by $k^+_{S_1}$ and $\bar{k}^+_{\bar{S}_1}$, respectively, represent the number of moles of a given species in the volume V, so

$$k^+_{S_1} = \bar{k}^+_{\bar{S}_1}. \quad (50)$$

Derivation of enzymatic reaction rates on platelet surfaces. Next, we derive rate constants for reactions between platelet-bound species. As an example, we focus on the formation of the complex C_1^b. As before, we introduce an equation which describes all species and reaction rates in a volumetric sense and another equation with bar variables that describes the same species and reaction rates on a surface. These equations are

$$\frac{dC_1^b}{dt} = \underbrace{k^+_{C_1}S_2^bE_1^b}_{\text{concentrations}} - (k^-_{C_1} + k^{\text{cat}}_{C_1})C_1^b, \quad (51)$$

and

$$\frac{d\bar{C}_1^b}{dt} = \underbrace{\bar{k}^+_{\bar{C}_1}\bar{S}_2^b\bar{E}_1^b}_{\text{surface densities}} - (\bar{k}^-_{\bar{C}_1} + \bar{k}^{\text{cat}}_{\bar{C}_1})\bar{C}_1^b, \quad (52)$$

respectively. As before, suppose that in volume V there are N^b bound platelets with $P^b\,V = N^b$. We then look at the rate at which the amount of C_1^b (in moles) changes over time, so then Equations (51) and (52) become

$$V\frac{dC_1^b}{dt} = k^+_{C_1}V\,S_2^bE_1^b - (k^-_{C_1} + k^{\text{cat}}_{C_1})V\,C_1^b, \quad (53)$$

and

$$N^b\, A_p\, \frac{d\bar{C}_1^b}{dt} = \bar{k}^+_{\bar{C}_1}\, \bar{S}_2^b\, \bar{E}_1^b\, N^b\, A_p - (\bar{k}^-_{\bar{C}_1} + \bar{k}^{\text{cat}}_{\bar{C}_1})\bar{C}_1^b\, N^b\, A_p, \tag{54}$$

respectively. In order to have the same rate transfer for the formation of C_1^b, then

$$k^+_{C_1}\, V\, S_2^b = \bar{k}^+_{\bar{C}_1}\, \bar{S}_2^b\, \bar{E}_1^b\, N^b\, A_p. \tag{55}$$

Note that the molar amount of E_1^b, S_2^b in a volume V can be written as

$$E_1^b V = N^b\, A_p\, \bar{E}_1^b, \qquad S_2^b V = N^b\, A_p\, \bar{S}_2^b, \tag{56}$$

respectively. Rewriting the terms in Equation (55), we find that

$$\frac{k^+_{C_1}}{V}\left(S_2^b\, V\right)\left(E_1^b\, V\right) = \bar{k}^+_{\bar{C}_1}\, \frac{\left(N^b\, A_p\, \bar{S}_2^b\right)\left(N^b\, A_p\, \bar{E}_1^b\right)}{N^b\, A_p}. \tag{57}$$

This equation necessitates that

$$\frac{k^+_{C_1}}{V} = \frac{\bar{k}^+_{\bar{C}_1}}{N^b\, A_p}, \tag{58}$$

Let $k^{+,s}_{C_1} = \bar{k}^+_{\bar{C}_1}$ and let $k^{+,v}_{C_1} = k^+_{C_1}$, then we have that

$$k^{+,s}_{C_1} = \frac{N^b}{V}\, k^{+,v}_{C_1}\, A_p, \tag{59}$$

and since $N^b = P^b\, V$, then

$$k^{+,s}_{C_1} = P^b\, k^{+,v}_{C_1}\, A_p. \tag{60}$$

Similarly, to have the same rate transfer for the disassociation and the catalytic conversion of C_1^b,

$$(k^-_{C_1} + k^{\text{cat}}_{C_1})\, V\, C_1^b = (\bar{k}^-_{\bar{C}_1} + \bar{k}^{\text{cat}}_{\bar{C}_1})\, \bar{C}_1^b\, N^b\, A_p. \tag{61}$$

Since $V\, C_1^b$ and $\bar{C}_1^b\, N^b\, A_p$ both represent the molar amount of C_1^b in a given volume, then

$$\bar{k}^-_{\bar{C}_1} = k^-_{C_1}, \quad \bar{k}^{\text{cat}}_{\bar{C}_1} = k^{\text{cat}}_{C_1}. \tag{62}$$

To summarize, we convert the volumetric coagulation reaction rates, $k^{+,v}_*$, to surface coagulation reaction rates, $k^{+,s}_*$, using the following relationships:

$$A_p = 4\pi(0.5 \times P_{\text{diam}})^2 \tag{63}$$

$$k^{+,s}_{C_1} = P^b \times k^{+,v}_{C_1} \times A_p \tag{64}$$

$$k^{+,s}_{C_2} = P^b \times k^{+,v}_{C_2} \times A_p \tag{65}$$

$$N_1^{\text{plt}} = 2700, \quad N_2^{\text{plt}} = 2000, \tag{66}$$

$$N_1 = N_1^{\text{plt}}/(N_A \times A_p) \tag{67}$$

$$N_2 = N_2^{\text{plt}} / (N_A \times A_p) \tag{68}$$

with $P^b = 5.51 \times 10^{13}$ platelet/dm^3, a characteristic number density of platelets in the aggregate, $A_p = 19.1$ μm^2 a characteristic platelet surface area, and $N_A = 6.022 \times 10^{23}$ mol^{-1} (Avogadro's constant). Here, $k_{C_1}^{+,v}$, $k_{C_2}^{+,v}$ are the volumetric reaction rates for the given reactions from [34].

For the subendothelium surface, we have binding and unbinding of fluid-phase species to E_0, but we have no reactions between pairs of subendothelium-bound species. Hence, there is no need to change additional subendothelium reaction rates to surface densities.

S3 Text. Fibrin polymerization model details. The model of fibrin polymerization is extended from [35], where here we allow fibrin monomers to advect and diffuse, and for fibrin monomer to be sourced from the enzymatic conversion of fibrinogen to fibrin by thrombin [36]. A fibrin cluster can be uniquely described by two indices, m and b, where b describes the number of branch points in an oligomer and $m + 2b$ is the total number of fibrin monomer in oligomer. Here, all species are are functions of spatial coordinates $\mathbf{x}$ and time t. With the reactions defined in Equations (8) and (9), the dynamic equations for $c_{mb}(\mathbf{x}, t)$ is

$$\begin{aligned}\frac{\partial c_{mb}}{\partial t} &= \delta_{m1,}\delta_{b,0}\big(\nabla\cdot(D\nabla c_{10}) - \mathbf{u}\cdot\nabla c_{10}(\mathbf{x},t)\big) + \delta_{m,1}\delta_{b,0}S_{mb} \\ &\quad \underbrace{+\frac{k_l}{2}\sum_{\substack{m_1+m_2=m\\ b_1+b_2=b}}(b_1+2)(b_2+2)c_{m_1b_1}c_{m_2b_2} - k_l(b+2)c_{mb}R}_{\text{link formation}} \\ &\quad \underbrace{+\frac{k_b}{6}\sum_{\substack{m_1+m_2+m_3=m+2\\ b_1+b_2+b_3=b-1}}(b_1+2)(b_2+2)(b_3+2)c_{m_1b_1}c_{m_2b_2}c_{m_3b_3} - \frac{k_b}{2}(b+2)c_{mb}R^2,}_{\text{branch formation}}\end{aligned} \tag{69}$$

where $\delta_{i,j}$ is the Kronecker delta. The Kronecker deltas multiplying the transport terms mean that only fibrin monomers, not dimers or larger oligomers move. The Kronecker deltas multiplying the source term S_{mb} means that only fibrin monomers are sourced. The source of monomers is from conversion of fibrinogen by thrombin and occurs at the rate

$$S_{10} = k_{\text{cat}}E_2\frac{G}{K_m+G}. \tag{70}$$

Here, G is the fibrinogen concentration, E_2 is the thrombin concentration, and R is the concentration of free reaction sites. The equations for c_{10}, G, and E_2 are

$$\frac{\partial c_{10}}{\partial t} = -\mathbf{u}\cdot\nabla c_{10} + D\Delta c_{10} + k_{\text{cat}}E_2\frac{G}{K_m+G} - (2k_lR + k_bR^2)c_{10}, \tag{71}$$

$$\frac{\partial G}{\partial t} = -\mathbf{u}\cdot\nabla G + D\Delta G - k_{\text{cat}}E_2\frac{G}{K_m+G}, \tag{72}$$

and

$$\frac{\partial E_2}{\partial t} = -\mathbf{u}\cdot\nabla E_2 + D\Delta E_2, \tag{73}$$

respectively, and the free reaction site concentration, R is defined as

$$R = \sum_{m_1,b_1}(b_1+2)c_{m_1,b_1}. \tag{74}$$

To obtain differential equations for the moments that define the species of interest, we introduce the moment generating function

$$g(\mathbf{x},t;y,z) = \sum_{m,b} y^m z^{b+2} c_{mb}(\mathbf{x},t). \tag{75}$$

Substituting this function into Equation (69), we obtain

$$\frac{\partial g}{\partial t} = \frac{k_l}{2} g_z^2 - k_l z g_z R + \frac{k_b}{6y^2} g_z^3 - \frac{k_b}{2} z g_z R^2 + P(\mathbf{x}, t; y, z) \tag{76}$$

Because only monomers move by advection and diffusion and only monomers are sourced,

$$P(\mathbf{x}, t; y, z) = yz^2 \left(-\mathbf{u} \cdot \nabla c_{10} + \nabla \cdot (D \nabla c_{10}) + k_{\text{cat}} E_2 \frac{G}{K_m + G} \right). \tag{77}$$

To reformulate the problem to study moments of the system, we define moments

$$M_{jk} = \left. \frac{\partial^{j+k} g(\mathbf{x}, t; y, z)}{\partial y^j \partial z^k} \right|_{y=1, z=1}. \tag{78}$$

These moments can be related to the quantities of interest. For example, the total concentration of free reaction sites, R, fibrin monomers in oligomer, θ, and branches B can expressed in terms of first moments as

$$R = \sum_{m,b} (b+2) c_{mb} = M_{01}, \tag{79}$$

$$\theta = \sum_{m,b} (m+2b) c_{mb} = M_{10} + 2M_{01} - 4M_{00}, \tag{80}$$

$$B = \sum_{m,b} b c_{mb} = M_{01} - 2M_{00}. \tag{81}$$

Using the PDE defined in Equation (76) and the relationship in Equation (78), we can then derive PDEs that describe how R, θ, and B change. First, we define equations for moments M_{00}, M_{10} and $M_{01} = R$, respectively:

$$\frac{\partial M_{00}}{\partial t} = -\frac{k_l}{2} R^2 - \frac{k_b}{3} R^3 - \mathbf{u} \cdot \nabla c_{10} + D \Delta c_{10} + k_{\text{cat}} E_2 \frac{G}{K_m + G}, \tag{82}$$

$$\frac{\partial M_{10}}{\partial t} = -\frac{k_b}{3} R^3 - \mathbf{u} \cdot \nabla c_{10} + D \Delta c_{10} + k_{\text{cat}} E_2 \frac{G}{K_m + G}, \tag{83}$$

$$\frac{\partial R}{\partial t} = -k_l R^2 - \frac{k_b}{2} R^3 + 2 \left(-\mathbf{u} \cdot \nabla c_{10} + D \Delta c_{10} + k_{\text{cat}} E_2 \frac{G}{K_m + G} \right). \tag{84}$$

From these equations, we can define equations for branch point density and fibrin monomer in oligomer as

$$\frac{\partial B}{\partial t} = \frac{k_b}{6} R^3, \tag{85}$$

and

$$\frac{\partial \theta}{\partial t} = -\mathbf{u} \cdot \nabla c_{10} + D \Delta c_{10} + k_{\text{cat}} E_2 \frac{G}{K_m + G}, \tag{86}$$

respectively.

Note that several equations above depend on the transport of fibrin monomer, c_{10}. For computational simplicity, we perform the following change of variables

$$Z = R - 2c_{10}, \qquad X = \theta - c_{10}, \tag{87}$$

where Z corresponds to the concentration of reaction sites in oligomers and X represents the concentration of monomers in oligomers.

As described in [35, 36, 61], the system of differential equations exhibits blow up in finite time, which we define as gelation. This can be interpreted as the emergence of an oligomer of infinite size, which is when the average oligomer size A, defined in Equation (13), becomes infinite in finite time. A can be expressed as a linear combination of moments of order zero, one, and two. A becomes infinite if and only if the specific second moment M_{02} or the related quantity $Y = M_{02} - R$ does so. The PDEs for M_{02} and Y are:

$$\begin{aligned}\frac{\partial M_{02}}{\partial t} &= k_l(M_{02}^2 - 2M_{02}R) + k_b(M_{02}^2 R - M_{02}R^2) \\ &\quad + 2\left(-\mathbf{u}\cdot\nabla c_{10} + \nabla\cdot(D\nabla c_{10}) + k_{\text{cat}}E_2\frac{G}{K_m+G}\right),\end{aligned} \tag{88}$$

and

$$\frac{\partial Y}{\partial t} = k_l Y^2 + k_b R\left(\frac{R^2}{2} + RY + Y^2\right). \tag{89}$$

As stated in the manuscript, the differential equations are not valid after gelation, the time that Y becomes unbounded at the spatial point in question. We define a binary gelation indicator variable, I, that indicates where gelation has occurred during the simulation. Once gelation occurs at a spatial point, x, we set $I = 1$ and all polymerization reactions stop so only advective and diffusive transport of fibrin monomer and fibrinogen can occur. We use the Kronecker delta in the polymerization terms to represent how gelation alters the dynamics of the following equations

$$\frac{\partial G}{\partial t} = -\mathbf{u}\cdot\nabla G + D\Delta G - k_{\text{cat}}E_2\frac{G}{K_m+G}, \tag{90}$$

$$\begin{aligned}\frac{\partial c_{10}}{\partial t} &= -\mathbf{u}\cdot\nabla c_{10} + D\Delta c_{10} + k_{\text{cat}}E_2\,\frac{G}{K_m+G} \\ &\quad - \delta_{I,0}\left\{2k_l(Z+2c_{10}) + k_b(Z+2c_{10})^2\right\}c_{10},\end{aligned} \tag{91}$$

$$\frac{\partial \theta}{\partial t} = -\mathbf{u}\cdot\nabla c_{10} + D\Delta c_{10} + k_{\text{cat}}E_2\frac{G}{K_m+G}, \tag{92}$$

$$\frac{\partial Z}{\partial t} = \delta_{I,0}\left\{-k_l(Z+2c_{10})^2 - \frac{k_b}{2}(Z+2c_{10})^3 + 2\left(2k_1(Z+2c_{10}) + k_b(Z+2c_{10})^2\right)c_{10}\right\}, \tag{93}$$

$$\frac{\partial B}{\partial t} = \delta_{I,0}\left\{\frac{k_b}{6}(Z+2c_{10})^3\right\}, \tag{94}$$

$$\frac{\partial X}{\partial t} = \delta_{I,0} \left\{ 2k_l(Z + 2c_{10}) + k_b(Z + 2c_{10})^2 \right\} c_{10} m \tag{95}$$

$$\frac{\partial Y}{\partial t} = \delta_{I,0} \left\{ k_l Y^2 + k_b (Z + 2c_{10}) \left(\frac{(Z + 2c_{10})^2}{2} + (Z + 2c_{10}) Y + Y^2 \right) \right\}. \tag{96}$$

S4 Text. Mesh and timestep convergence study Preliminary simulations are performed with the loose plug at a shear rate of 1000 s^{-1} to check for time step and mesh independence. Three meshes are generated for these tests, consisting of coarse, medium, and fine refinement levels. The meshes have 11396, 29463, and 72051 computational cells, respectively. To assess temporal sensitivity, simulations were repeated on the fine mesh using time-step sizes ranging from 1 $\times 10^{-2}$ to 5 $\times 10^{-4}$ s, keeping all other parameters constant. To evaluate spatial sensitivity, simulations were repeated on the three mesh refinement levels, keeping all other parameters constant with a time-step size of 1 $\times 10^{-3}$s. The resulting spatiotemporal development of key coagulation species was compared across these simulations to verify time-step and mesh independence. The maximum concentration and total amount of thrombin (E_2), monomers (c_{10}), and monomers in oligomers (θ), and the gel-covered area are measured at three time points: before gelation ($T = 30$ s), directly after the gelation-onset ($T = 45$ s), and after gelation finalization ($T = 60$ s). Following the method from [64], we determined the spatial discretization error as the grid convergence index. In this method, Q_3, Q_2, Q_1 are the quantities on the coarse, medium, and fine mesh, respectively, and the effective refinement ratio r is estimated as $r_{23} \approx \sqrt{N_2/N_3}$, and $r_{21} \approx \sqrt{N_1/N_2}$, in which N_i is the number of elements per mesh. The relative errors are $e_{21} = |(Q_1 - Q_2)/Q_1|$ and $e_{32} = |(Q_2 - Q_3)/Q_2|$. With safety factor $F_s = 1.25$, and order p=2, the GCIs are

$$\mathrm{GCI}_{21} = \frac{F_s\, e_{21}}{r_{21}^p - 1}, \qquad \mathrm{GCI}_{32} = \frac{F_s\, e_{32}}{r_{32}^p - 1},$$

We consider the mesh as spatially independent when GCI $< 5\%$.

Temporal sensitivity on the fine mesh was quantified by successive percent changes between time-step levels $\Delta t_k \to \Delta t_{k+1}$:

$$\Delta_k = \frac{|Q(\Delta t_{k+1}) - Q(\Delta t_k)|}{|Q(\Delta t_{k+1})|} \times 100\%$$

We consider the time-step to be converged when $\Delta_k < 3\%$.

The three-level spatial convergence study in S1 Fig shows that for all seven output metrics at $T = 30\,\mathrm{s}$, $T = 45\,\mathrm{s}$, and $T = 60\,\mathrm{s}$ the fine-grid GCI values are below 5%. Therefore the medium mesh is sufficient, reducing computational cost by $\sim$60% compared to the fine mesh. The temporal convergence study in S2 Fig indicates that the change in the seven output metrics between $\Delta t = 1 \times 10^{-3}$ s and $\Delta t = 5 \times 10^{-4}$ s is below 3% in all cases. Thus a time-step of $\Delta t = 1 \times 10^{-3}$ s is sufficient for capturing the coagulation dynamics without significant temporal discretization error. Therefore, the medium mesh and a time step size of 1×10^{-3} s are adopted for the final simulations.

S1 Fig. Spatial sensitivity of coagulation variables for different mesh resolutions. Spatial sensitivity of key coagulation variables at $\Delta t = 10^{-3}$ s for three mesh resolutions (coarse, medium, fine). Curves show results at $T = 30, 45, 60$ s with circular markers at each mesh configuration. The grid convergence index (GCI) is indicated for each panel, where the segment between Coarse→Medium is annotated with GCI_{32} and the segment between Medium→Fine with GCI_{21} (both in %). Panels are arranged by quantity: top row—maximum values (thrombin, monomers in oligomers, monomers); middle row—total domain-integrated values (thrombin, monomers in oligomers, monomers); bottom row—gel-covered area.

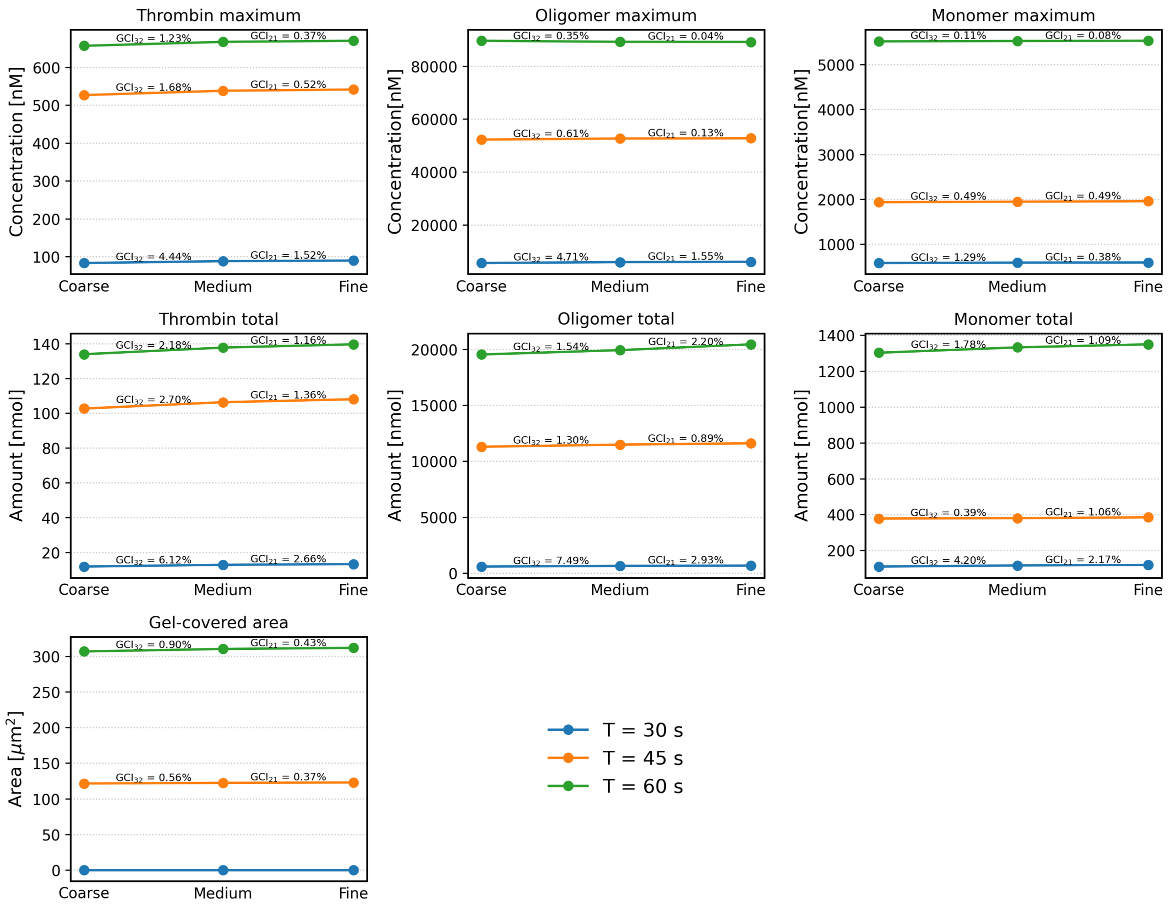

S2 Fig. Temporal sensitivity of coagulation variables on the fine mesh at different time points. Temporal sensitivity of key coagulation variables on the fine mesh at three analysis times ($T = 30$, 45, 60 s). Each subplot shows the variable versus the time step ($\Delta t = 0.01 \to 0.005 \to 0.001 \to 0.0005\,$s), with Δt on a logarithmic x-axis. Curves correspond to the four time points, with circular markers at the tested Δt levels. The successive percent changes between adjacent Δt levels are indicated. Panels are arranged by quantity: top row—maximum values (thrombin, monomers in oligomers, monomers); middle row—total domain-integrated values (thrombin, monomers in oligomers, monomers); bottom row—gel-covered area.

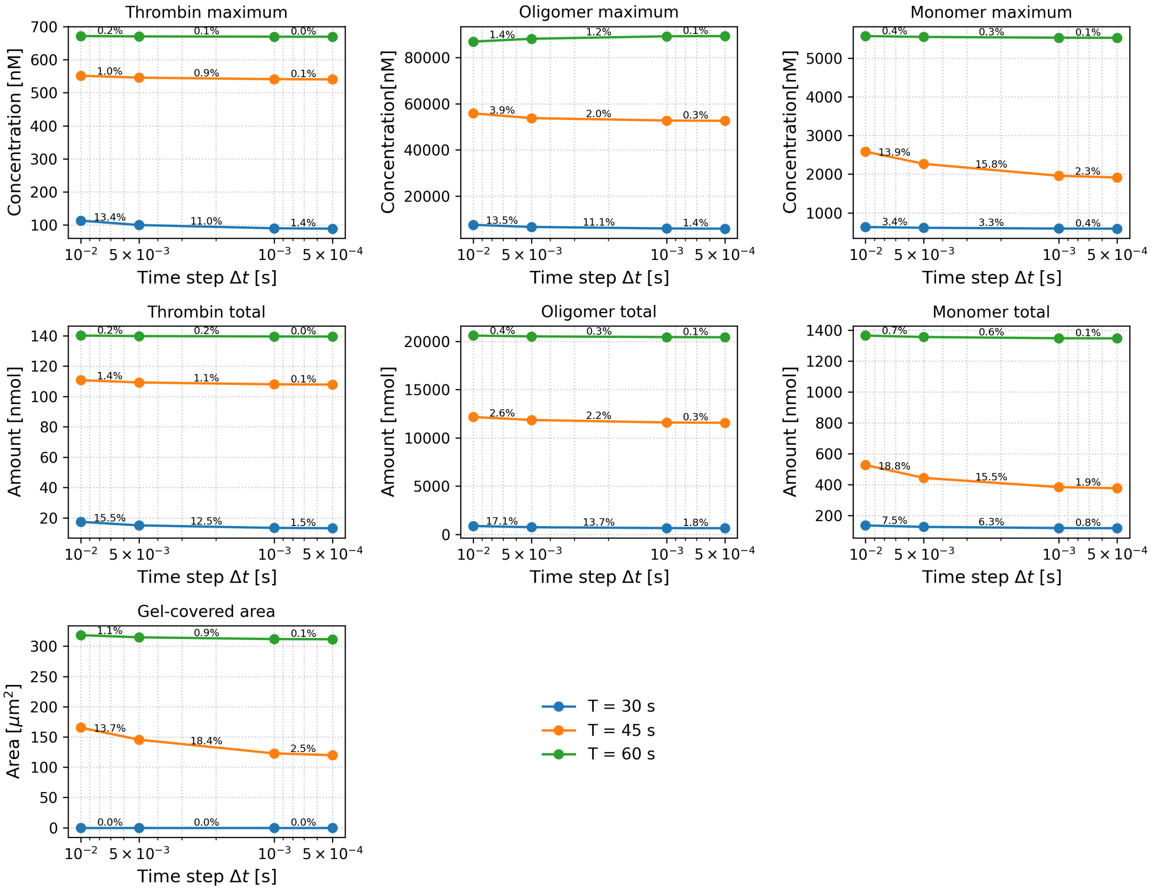

S3 Fig. Branching rate estimation from data in [40]. To estimate k_b in Fibrin polymerization model, we simulate the original, zero-dimensional polymerization model in [35] with a Michaelis–Menten source term for fibrin monomer that depends on the thrombin and fibrinogen concentrations. For different combinations of thrombin and fibrinogen concentrations and a fixed branching rate, k_b, the fibrin polymerization model will output the gel time, t_{gel} which corresponds to the finite time blow up of M_{02} and Y. We compare these computational gel times (color lines) to experimental gel times (red dots), where thrombin is varied and fibrinogen is fixed at 3 mg/mL [40]. We find that $k_b = 1.5 \times 10^9$ $M^{-2}s^{-1}$ best approximates the behavior seen in experiments [40].

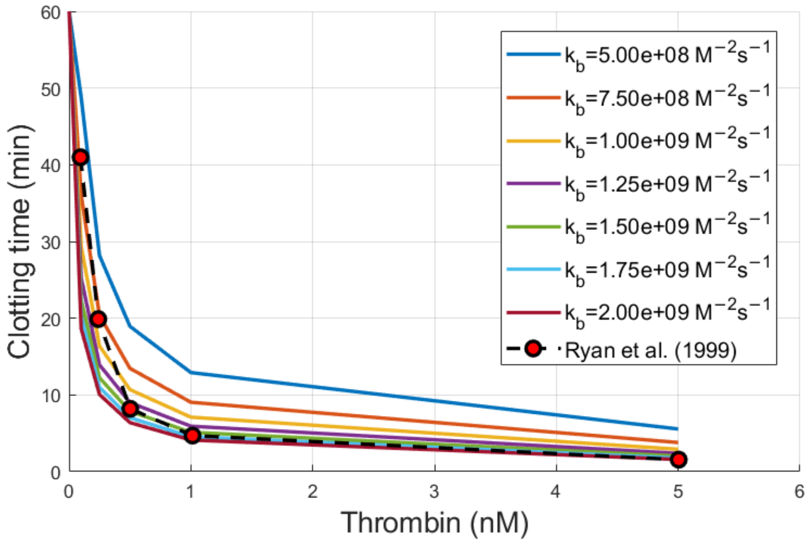

S4 Fig. Fibrin gel area over time for different platelet plug configurations. Time evolution of the fibrin gel area is shown for different platelet plug configurations (color) and different shear rates (line style) for up to 120 s.

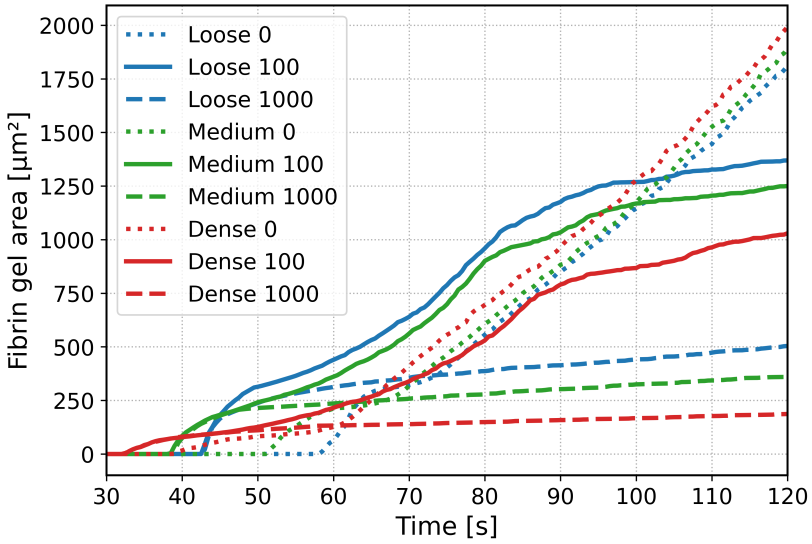

S5 Fig. 2D plots of fibrin polymerization species concentrations. Concentrations of fibrin polymerization species in the fluid for (A) the loose platelet plug and (B) the dense platelet plug with shear rate 1000 s^{-1}. Each row corresponds to a different time value, the color bar for each column refers to the concentration, and the horizontal bar represents 10 μm.

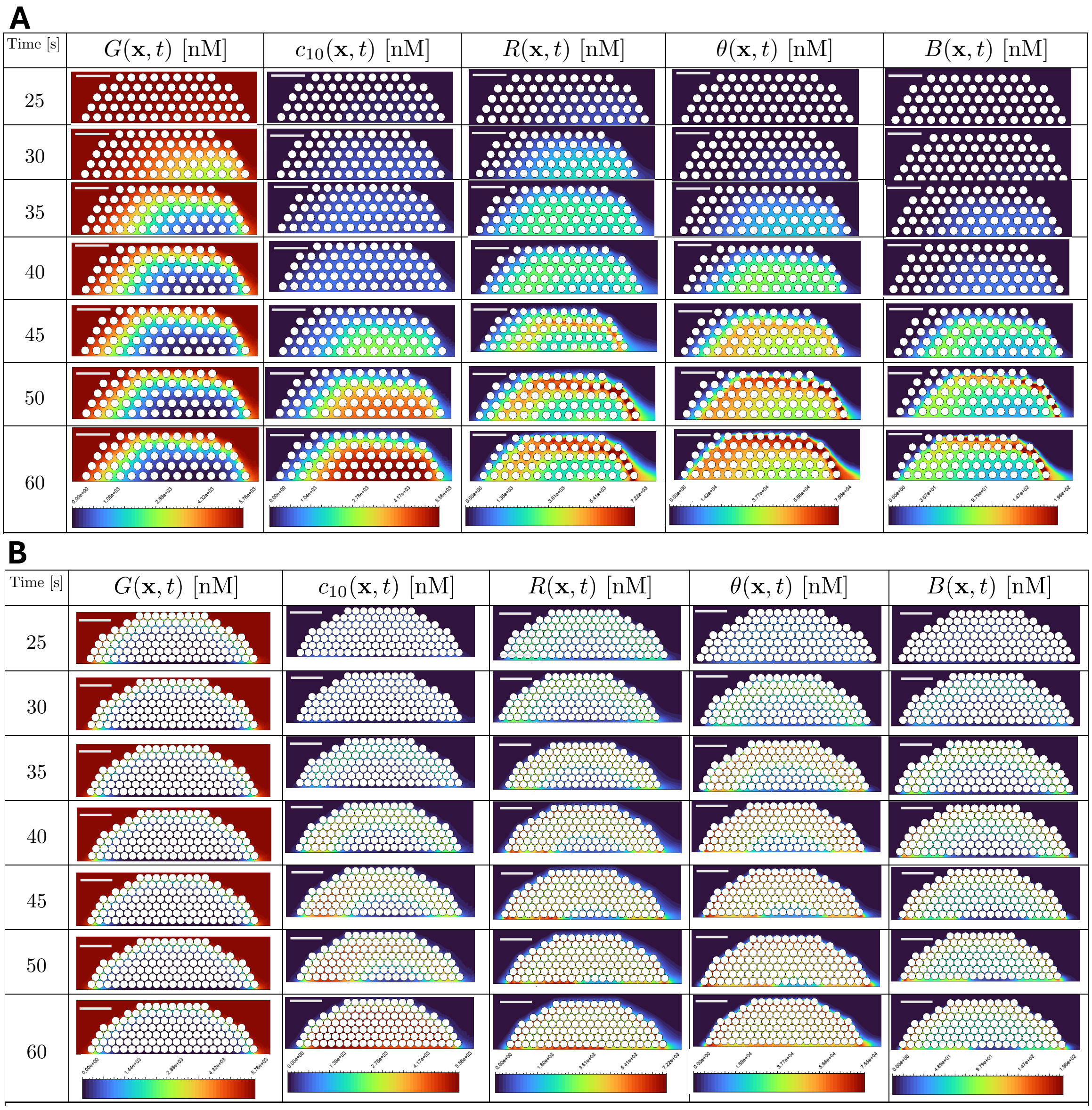

S6 Fig. 2D plots of fibrin transport and production rates. Transport (concentration per time) of fibrinogen $G(\mathbf{x}, t)$ and transport and production (concentration per time) of fibrin monomer $c_{10}(\mathbf{x}, t)$ over the domain around the platelet plug for (A) the loose platelet configuration and (B) the dense platelet configuration for shear rate $\dot{\gamma} = 1000\ \mathrm{s}^{-1}$. Positive and negative values indicate transport into and out of a point in the domain, respectively. Horizontal bar corresponds to 10 μm.

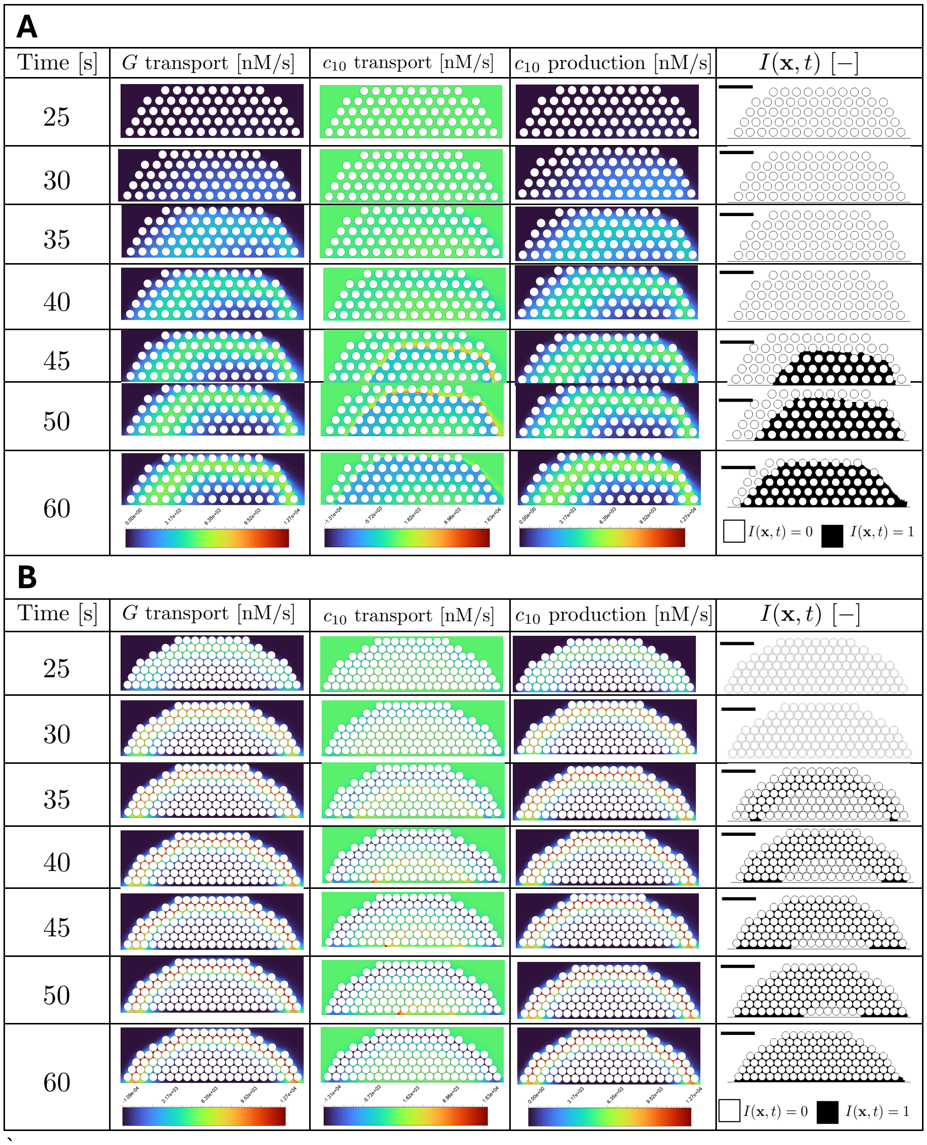

S7 Fig. Flow comparison for three spatial points for coagulation species and fibrinogen. Time courses of coagulation species and fibrinogen concentrations for the loose platelet plug with (a) flow and (b) without flow and for the dense platelet plug with (c) flow and (d) without flow. Each color refers to a spatial location within the platelet plug: high point (red), middle point (cyan), and low point (blue). For each simulation, the dot corresponds to the time of gelation. Note that if there is no marker, gelation does not occur before 60 seconds.

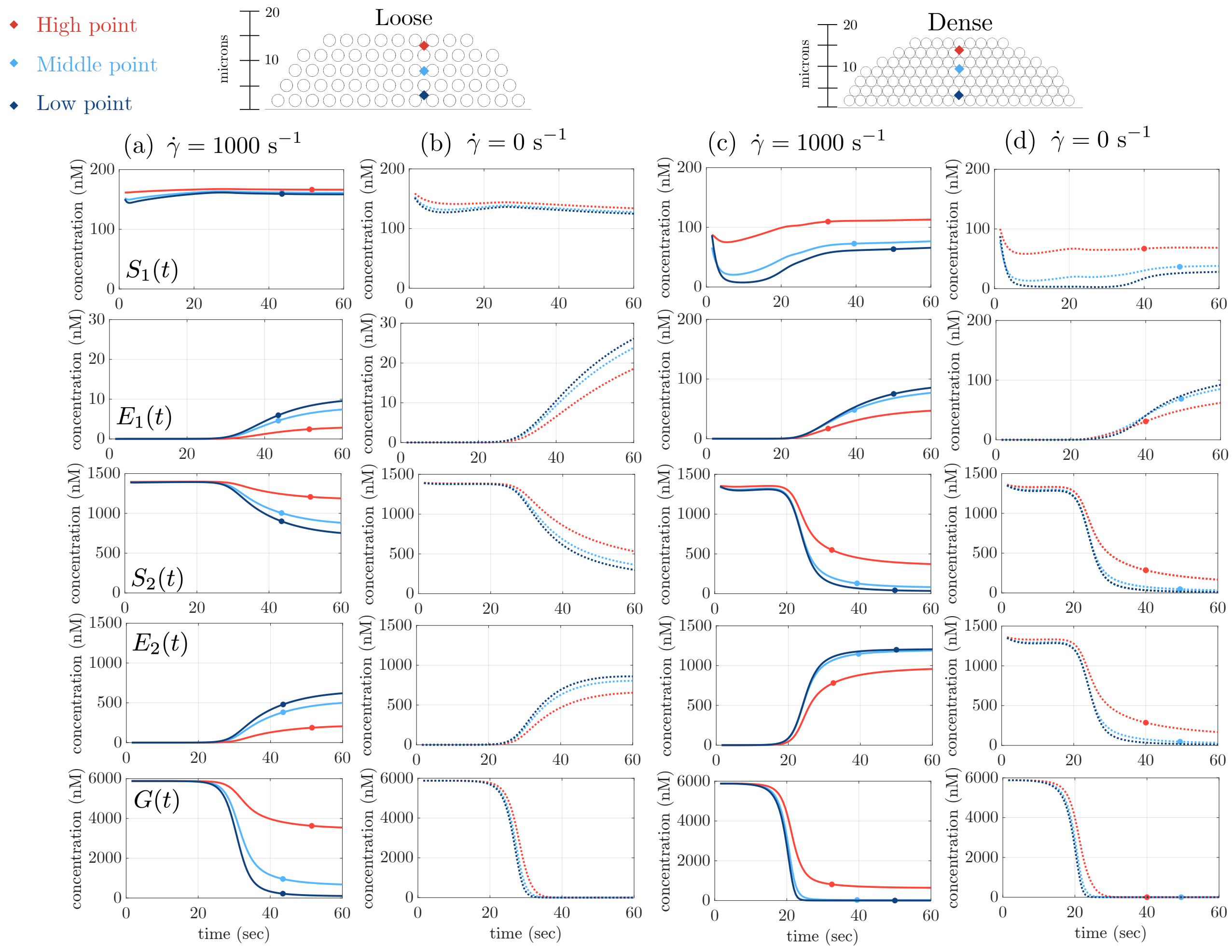

S8 Fig. Flow comparison for three spatial points for fibrin polymerization species. Time courses of fibrin polymerization species for the loose platelet plug with (a) flow and (b) without flow and for the dense platelet plug with (c) flow and (d) without flow. Each color refers to a spatial location within the platelet plug: high point (red), middle point (cyan), and low point (blue). For each simulation, the dot corresponds to the time of gelation. Note that if there is no marker, gelation does not occur before 60 seconds.

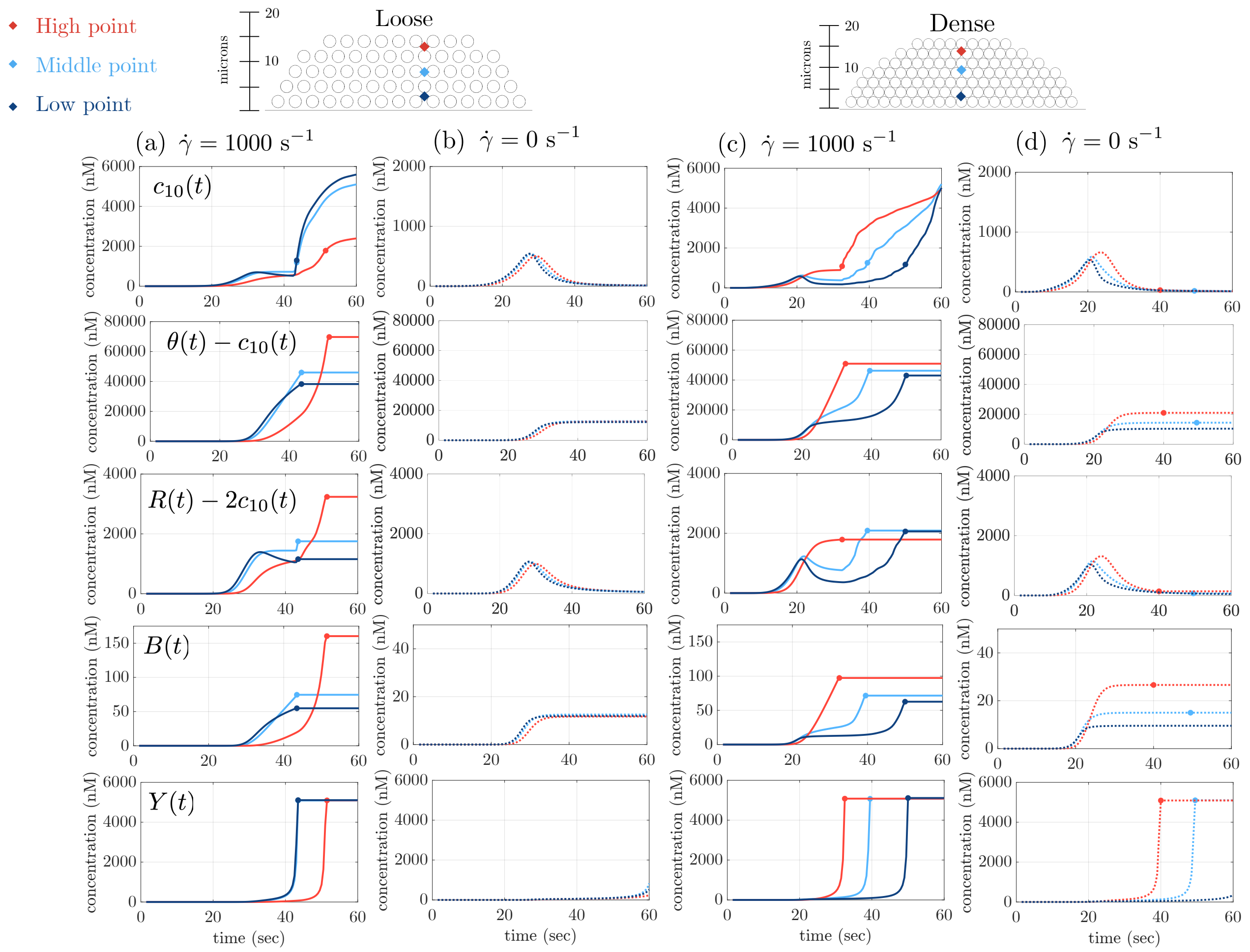

S9 Fig. Flow comparison for three spatial points for transport, consumption, and production rates. Rates of transport, consumption, and production for the loose platelet plug with (a) flow and (b) without flow conditions and for the dense platelet plug with (c) flow and (d) without flow. Each color refers to a spatial location within the platelet plug: high point (red), middle point (cyan), and low point (blue). For each simulation, the dot corresponds to the time of gelation. Note that if there is no marker, gelation does not occur before 60 seconds.

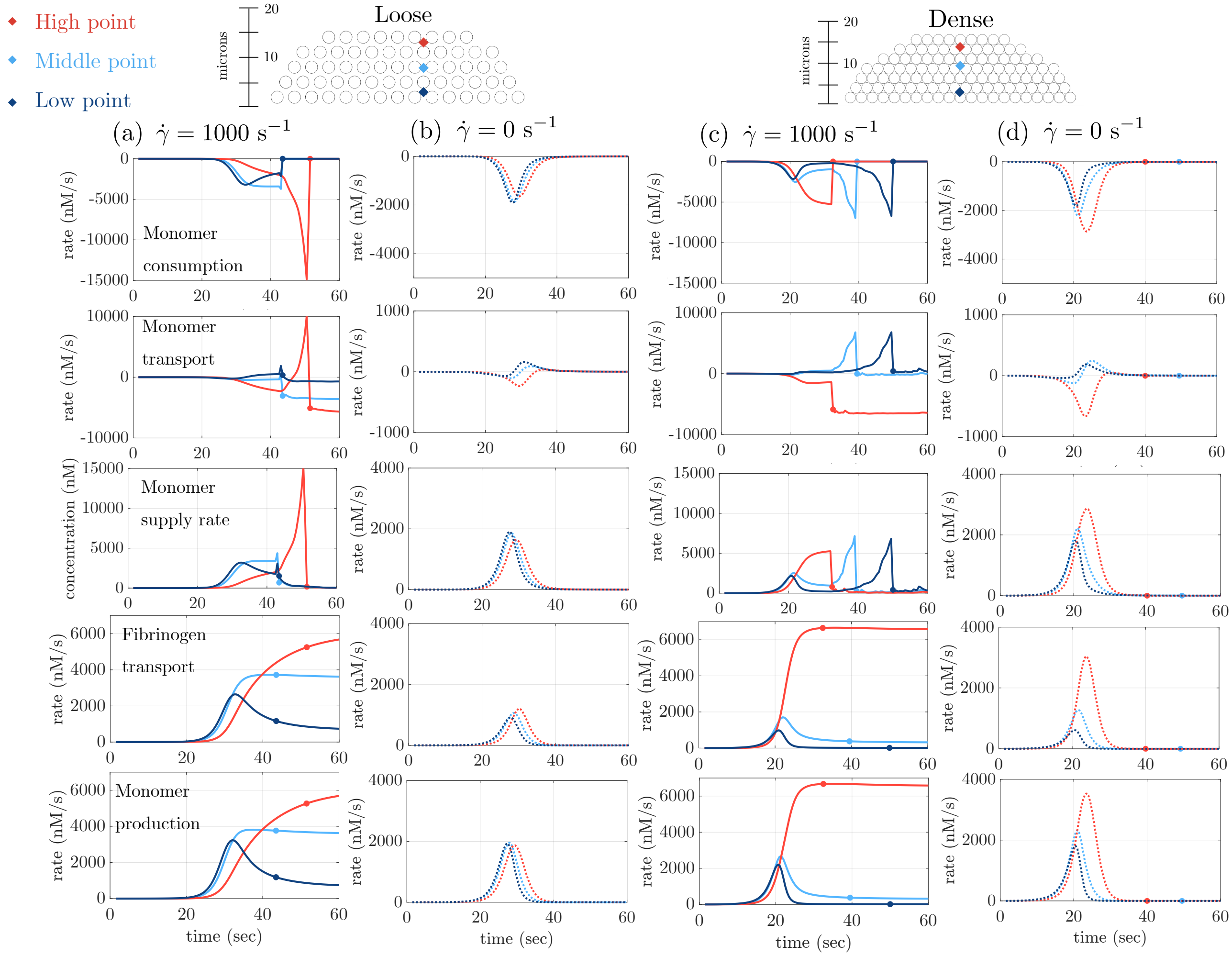

S10 Fig. Comparison of PDE model results with ODE model adapted from [35]
Comparison of PDE results at the low point in the dense platelet plug with results from a zero-dimensional ODE model of fibrin polymerization in [35]. With the modification, the source of fibrin monomer is fibrinogen converted to fibrin monomer by thrombin rather than a steady rate of supply as in [35]. In the modified ODE model, we use the time courses of fibrinogen G and thrombin E_2 found in the PDE framework at that spatial location. Left: Concentrations of fibrinogen and thrombin from the PDE model at the low point in the dense platelet configuration with $\dot{\gamma} = 1000\ \mathrm{s}^{-1}$. Right: Comparison of results from the full, PDE model (solid line) to zero-dimensional ODE model (dotted line) results from a modification of [35] with $G(t)$ and $E_2(t)$ from the PDE model as inputs. The dot in both panels refers to the gel time for the low spatial location for the PDE model, and vertical line refer to first gel time at the high point.

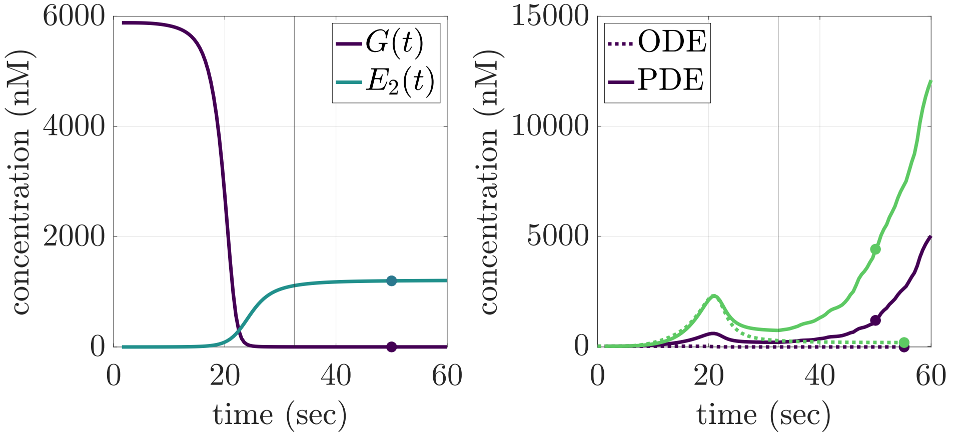

Supplement: Supplement 1 [file NIHPP2604.07844v1-supplement-1.pdf]
